# Temperature and pH dynamics during carcass decomposition and implications for disease management

**DOI:** 10.1038/s41598-025-07716-w

**Published:** 2025-07-16

**Authors:** Janine Rietz, Burkhard Beudert, Nicolas Ferry, Lukas Böcker, Franz J. Conraths, Carolina Probst, Andreas Zedrosser, Helmut Küchenhoff, Martin Hais, Jens Schlüter, Tomas Lackner, Christian von Hoermann, Jörg Müller, Marco Heurich

**Affiliations:** 1 Department of National Park Monitoring and Animal Management, Bavarian Forest National Park, Grafenau, Germany; 2 Chair of Wildlife Ecology and Management, Albert-Ludwigs-University Freiburg, Freiburg, Germany; 3 Department of Conservation and Research, Bavarian Forest National Park, Grafenau, Germany; 4 Clinical unit, Small Animal Surgery, Department of Companion Animals and Horses, University of Veterinary Medicine Vienna, Vienna, Austria; 5 Friedrich-Loeffler-Institut, Institute of Epidemiology, Greifswald – Insel Riems, Germany; 6 Present Address: Federal Ministry for Economic Cooperation and Development, Berlin, Germany; 7 Department of Integrative Biology, Institute of Wildlife Biology and Game Management, University of Natural Resources and Applied Life Sciences, Vienna, Austria; 8 Department of Natural sciences and Environmental Health, University of South- Eastern Norway, Bø i Telemark, Norway; 9 Department of Statistics, Statistical Consulting Unit StaBLab, Ludwig- Maximilians-Universität, Munich, Germany; 10 Department of Ecosystem Biology, Faculty of Science, South Bohemian University, České Budějovice, Czech Republic; 11 Department of Environmental Systems Science, ETH Zürich, Zürich, Switzerland; 12 Department of Animal Ecology and Tropical Biology, University of Würzburg, Würzburg, Germany; 13 Faculty of Applied Ecology, Agricultural Sciences and Biotechnology, University of Inland Norway , Koppang, Norway

**Keywords:** African swine fever, Carcass decomposition, Carcass temperature, pH, Wild Boar, Wildlife diseases, Ecology, Ecological epidemiology

## Abstract

Infectious diseases in wildlife threaten not only those species but also domestic animals and human health, necessitating strategies to prevent pathogen spread. The natural decomposition of carcasses may lead to pathogen inactivation due to associated increases in temperature and changes in the pH of the carcass and in the surrounding soil. In this study, the internal temperatures of 64 decomposing wild boar carcasses, the pH in the topsoil beneath 74 carcasses, and the pH of muscle and rectal tissue from 12 carcasses were monitored throughout the decomposition process. Carcass temperatures increased during decomposition, frequently exceeding 30 °C during aerobic decomposition in summer (maximum 58 °C). The pH in the carcasses increased until skeletonization, ranging from pH 4 to above pH 8. Soil pH also continuously increased during the decomposition, reaching a pH above 9 and remaining stable for at least 30 days post-skeletonization. The information on natural carcass decomposition processes provided by our study can serve as a basis for future studies to assess if elevated carcass temperatures and pH changes are sufficient for pathogen inactivation. However, our results suggest that, in most cases, neither the increase in carcass temperatures nor the changes in pH exceed the thresholds required to inactivate African swine fever virus.

**Supplementary Information:**

The online version contains supplementary material available at 10.1038/s41598-025-07716-w.

## Introduction

Emerging infectious wildlife diseases pose significant threats not only to wildlife populations and domestic animals but also to humans, via zoonotic pathogens^1–3^. Anthropogenic impacts on nature, including habitat degradation, human encroachment into previously untouched habitats, biological invasions, climate change, and biodiversity loss, increase the risk of infectious disease transmission between wildlife, domestic animals, and humans^1^. In addition, altered forms of land use, including increasing crop production, changes in hunting practices, and warmer winters have increased the size and range of several wildlife species populations in Europe and North America, such as wild boar^4,5^. This development, coupled with the emergence of previously unknown pathogens, has led to growing concern regarding disease transmission for diseases such as African swine fever (ASF) and avian influenza^2^.

Research into the role of wildlife in disease transmission has shown that wildlife species can serve as maintenance hosts, contributing to the persistence and spread of pathogens both within their own populations and in domestic animals^3^. Wild boar play a significant epidemiological role, as they are well-connected to other species by indirect interactions^6^and are primary hosts for pathogens such as hepatitis E virus, influenza A virus, Salmonellae, Aujeszky’s disease virus, *Brucella* spp. and mycobacteria^7,8^. Both Classical swine fever virus (CSFV) and African swine fever virus (ASFV) may further be transmitted by wild boar^9^.

ASF is a viral disease originating in Africa that affects both wild boar and domestic pigs. The disease has recently spread across Europe, Asia, and the Caribbean^10^. Its impact has been profound, as it has resulted in significant economic losses in domestic pig industry and dramatic declines in affected wild boar populations^11,12^. The causative agent, ASFV, is known for its tenacity, in part due to its temperature resistance and lack of pH sensitivity^13,14^. Carcasses of wild boar that have died from ASFV can remain infectious for several months^15–17^and ASFV DNA has been found in soil at contaminated carcasses^18^. Carcasses are thus a major factor in the transmission of ASFV within wild boar populations^19,20^as the direct and indirect contact of wild boar with infected carcasses, such as by chewing on bones or rooting in the surrounding soil, can result in disease spread^11,18,21^. Carcasses can also promote the spread of other pathogens, such as pathogenic *E. coli* and *Salmonella* spp., and may thus pose health risks to animals through direct (scavenging) and indirect contact (rooting in the surrounding soil)^22,23^.

Pathogen stability and infectivity depend on various factors such as the type of tissue (e.g. bones, muscle, skin, fat), the surrounding medium (e.g., different soil types, water), and the weather conditions (sunlight exposure, temperature, humidity)^16,24^. Some pathogens remain infectious at carcass sites for long periods of time. For example, the prions responsible for scrapie persist in the soil for at least 29 months^25^while those causing transmissible spongiform encephalopathy can remain in the environment for several years or even decades. The environmental persistence of the bacterium *Bacillus anthracis*, the causative agent of anthrax, is also well established^26,27^. Thus, in terms of wildlife disease management, the effective elimination of these pathogens is a continuing challenge^28,29^and various methods for their elimination have been tested^30^. One possible approach could be inactivation during the natural carcass decomposition, to which the activities of enzymes, microbes (bacteria and fungi), invertebrates, and vertebrate scavengers contribute^31^.

The decomposition process consists of distinct stages, transitioning from anaerobic putrefaction to active, aerobically mediated decay, often facilitated by the activity of fly maggots opening the skin^32^. Fly eggs are oviposited during the early stages of carcass decomposition, often accelerating the degradation process towards active decay^32^. The resulting maggot masses feed on the carcass body tissue, thereby generating metabolic heat^33–35^which can exceed the ambient temperature by more than 20 °C^36,37^.

Wildlife pathogens differ in their temperature sensitivity such that their activity may decline below or above certain thresholds. For example, temperatures exceeding 37 °C are required to reduce the infectivity of avian influenza virus to undetectable levels whereas the threshold for hepatitis E virus is 60 °C^38,39^. ASFV can persist for several months within carcasses both at room temperature and at − 20 °C^16^ whereas at temperatures above 53 °C it is rapidly inactivated^14,40^.

In addition to temperature sensitivity, pathogens might be influenced by changing pH levels such as those that occur during decomposition. The chemical processes that cause cellular breakdown in the carcass result in the release of fatty and amino acids, which alter the internal carcass pH and subsequently the soil pH^41^. Most studies have reported an increase in the pH of the soil beneath decomposing carcasses^42–44^ followed by a decline due to the production of salpetric acid via microbial nitrification during the later stages of decay^45,46^. Although several studies have investigated the effect of carcass decomposition on soil pH, the pH changes within carcasses have been examined in only a few studies, mainly in relation to meat and food production^47^blood^48^or larval masses in decomposing carcasses^49^. However, pathogens vary in their resistance to pH changes. ASFV, for example, remains infectious over a pH range from pH 4 to 9 or even 13^14,50^.

Whether carcass decomposition in natural habitats reaches critical temperature and pH values necessary for pathogen inactivation is unknown. To the best of our knowledge, no study has measured the changes in pH that occur in carcasses in situ, and specifically in different body parts over the complete decomposition process. Additional research on carcass temperatures and pH development, including in the soil beneath the carcass, is needed to better understand disease dynamics, especially for pathogens with the potential to cause a pandemic, such as ASFV. Thus, in this study we investigated (1) the temperature and (2) the pH dynamics of decomposing wild boar carcasses, based on measurements taken in the muscle and in the rectum, and (3) assessed the pH changes in the topsoil beneath the carcasses at regular intervals during the decomposition process.

## Methods

### Study area

The study was conducted in the Bavarian Forest National Park (BFNP, 249 km^2^49° 3′ 19″N, 13° 12′ 9″E), located in south-eastern Germany and extending over an elevation range of 715–1105 m.a.s.l. The annual air temperature varies between 3.5 and 7.2 °C, and annual precipitation between 830 and 1820 mm. Snow cover is present in winter and typically peaks from January to March^51,52^. The area is covered by temperate forests composed mainly of Norway spruce (*Picea abies*), European beech (*Fagus sylvatica*), and silver fir (*Abies alba*)^53^. Soils (according to WRB (2022)) are mainly dystric cambisols with varying degrees of podsolization, gleysols, and histosols, and to a lesser extent leptosols. The pH of the mineral topsoil (0–5 cm) is around 3.1 (KCl) and 3.9 (H_2_O), without any significant change since 1990 (Beudert et al. 2023, unpublished).

### Experimental design

**Fig. 1:**
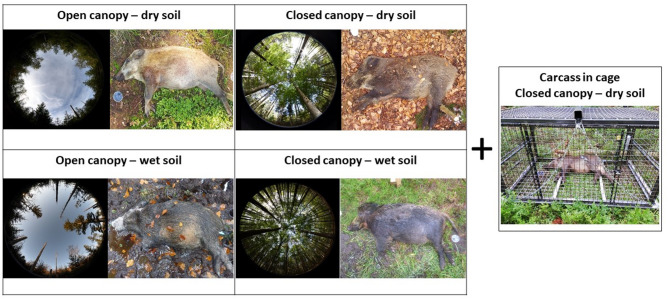
Experimental setup of a placement series: Two carcasses were placed in an open canopy environment, one on dry soil and the other on wet soil. Similarly, two carcasses were placed under a closed canopy, on dry and wet soils. During the warmer season, one carcass was additionally enclosed within a cage to exclude vertebrate scavenger activity.

Between July 2020 and 2021, we placed 74 wild boar carcasses in the BFNP (four carcasses every 3 weeks, 16 placement series in total). During warmer seasons, one additional carcass was exposed in a closed cage to exclude vertebrate scavenger activity (*n* = 10). Within each placement series, two carcasses were placed in open-canopy habitats (meadows or deadwood areas), and two others in forests (*n* = 32 each). Within each habitat type, one carcass was positioned on dry soil and the other on wet soil, based on our assessment (*n* = 32 each). Carcasses in cages were placed in closed forests on dry soil. The experimental setup is shown in Fig. 1. Each site was situated at least 500 m apart from the others. The carcasses were from wild boar shot as part of the regular wildlife management of the BFNP; the animals tested negative for ASFV and Aujeszky’s disease (using PCR and ELISA, respectively). Their weights ranged from 9 kg (female piglet) to 95 kg (adult male). The non-eviscerated carcasses were frozen at − 20 °C until 1–4 days before placement, depending on their weight, to allow them to thaw completely before being placed in the environment. One wild boar was shot in the morning of each planned placement series (*n* = 16 in total) and used as one of the four carcasses alternatingly placed in the tested habitat type and soil condition to examine differences in decomposition between previously frozen and not frozen carcasses, as freezing could damage tissues and hamper internal bacterial activity^54^. To prevent vertebrate scavengers from relocating carcasses at the placement sites, the hind limbs were securely fastened to fixed poles using cable ties. All experiments were performed in accordance with relevant guidelines and regulations.

Due to high snow cover, cold temperatures causing frozen soil, and the absence of visible carcass decomposition, we suspended carcass placements in January and February 2021. Consequently, temperature and pH measurements were limited during those months.

**Carcass temperature** was measured in situ in 64 carcasses using rectally inserted temperature loggers (iButton MF1921G, Maxim Integrated, CA, USA) set to record at 30-min intervals beginning from the placement day until carcass skeletonization (i.e., dry remains with only bones and skin left). Simultaneously, air temperature was recorded at a distance of 2 m from the carcass and a height of 15 cm above the ground using TOMST data loggers (TOMST TMS-4, Prague, Czech Republic) (Wild et al. 2019). **Carcass pH measurements** were conducted from July to December 2020 and from June to August 2021, with 16 trials in 11 carcasses. pH probes were inserted into the hind thigh musculature or rectum, with eight trials for each measurement method (Figure S1). A PHM 230 m (Dostmann Electronic GmbH, Wertheim-Reicholzheim, Germany), equipped with a PHE04 probe (datenlogger-store, Eichstetten, Germany) designed to use in soft and solid media, was used for continuous pH and temperature recordings at 30-min intervals. Data loggers were controlled on-site every second or third day. The probes were then calibrated using pH 4 and 7 buffer solutions and inserted at the previous position. We removed the pH meters once a carcass had reached the early dry remains stage (onset of skeletonization), with only bones and skin with hairs remaining, and when the pH probe became exposed (Supplementary Figure S1). **Soil pH** was assessed by collecting topsoil (organic layer 0–3 cm) directly beneath each carcass abdomen (midpoint) using a handheld soil sampler (Fiskars QuickDrill 150 mm, Fiskars Group, Espoo, Finland). Soil was collected from all 74 wild boar carcass placement sites. The first soil sample was collected directly before carcass placement to obtain a reference sample of the soil pH unaffected by the presence of the carcass. Sampling varied from 8 to 17 days depending on the season, decomposition rate, and decomposition stage, with routine sampling on days 0, 13 or 16, 30 and day 60. If decomposition was incomplete (skeletonization not yet reached) by day 60 or when winter conditions inhibited morphologically evident decomposition, sampling was extended up to 210 days (Supplementary Figure S2). For each series, the carcass in the cage and one additional carcass, alternatingly placed on dry or wet soil, were sampled more frequently, i.e., at days 0, 2, 4, 6, 9, 13, 16, 23, 30, and 60, or until skeletonization was reached. For details, see Table S1 in the Supplementary Information. Soil samples were mixed with distilled H_2_O (1:5) and allowed to rest for 20–24 h, according to DIN EN ISO 10390:2022. pH levels were measured using a MultiLine^®^ Multi 3510 IDS pH-meter equipped with a pH-E BlueLine 14 pH-probe (WTW & SI Analytics, respectively; Xylem Analytics Germany Sales GmbH & Co. KG, Weilheim, Germany), which includes automatic temperature compensation, as well as pH buffer solutions (pH 4 and pH 7) for calibration.

### Environmental variables and carcass conditions

**Fig. 2:**
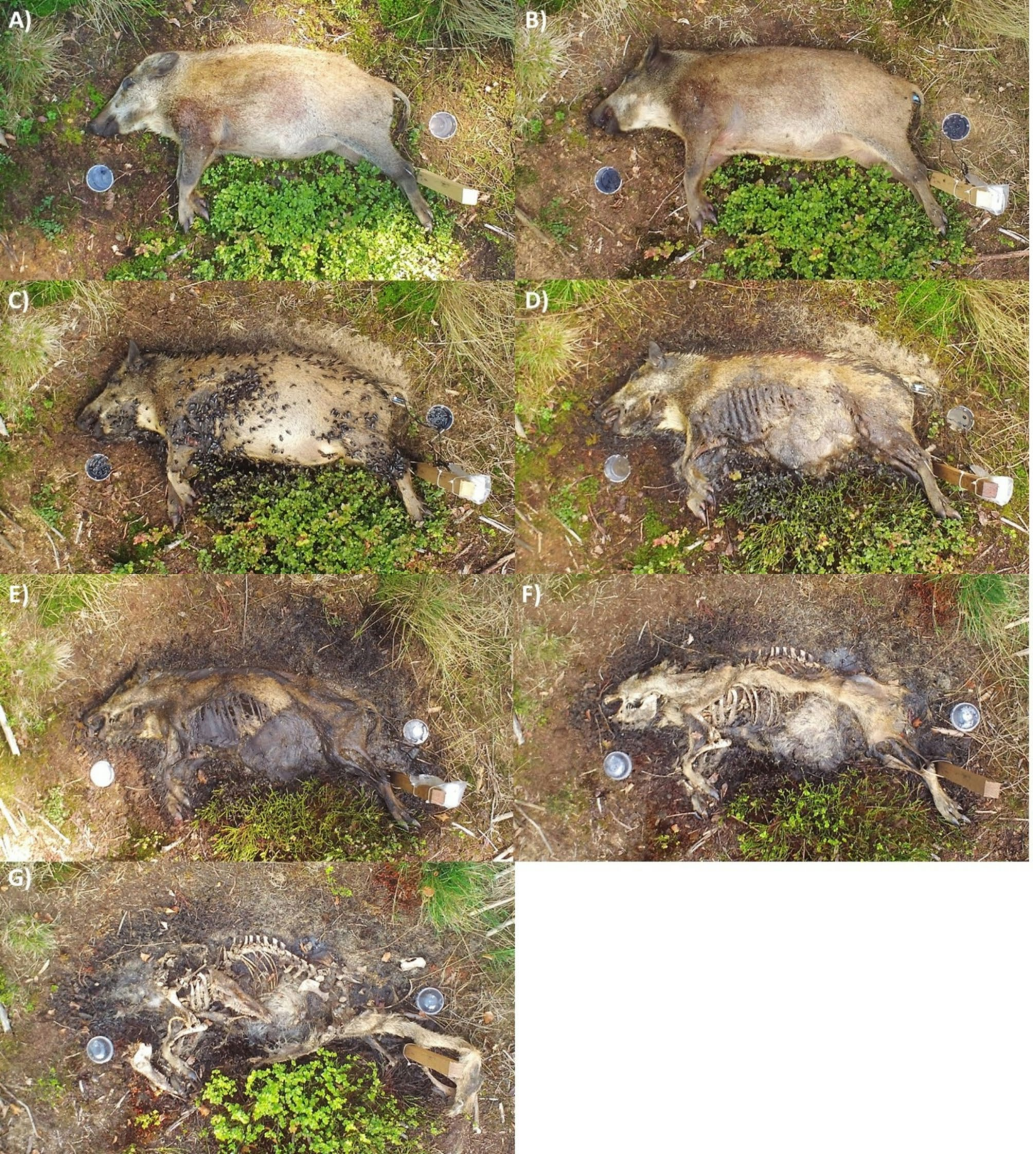
Decomposition of a wild boar in an open habitat on dry soil during August and September 2020. The hind leg of the carcass was tied to a fixed wooden pole to prevent vertebrate scavengers from moving the carcass outside the camera view. A pH-meter was also fixed to the pole, with the pH- and temperature probes inserted rectally in the carcass. **(A)** Day of carcass placement: The previously frozen carcass was thawed for 2 days before placement. Putrefaction has started, with visible changes in skin color (reflecting sulfhemoglobin formation). **(B)** Bloated stage (Day 1): Visible inflation of the abdomen. **(C)** Bloated stage (Day 4): Necrophagous beetle species (Nicrophorus littoralis) opened the abdomen, which has started to deflate. **(D)** Post-bloating (Day 7): Strong leakage of decomposition fluids. **(E)** Advanced decay stage (Day 13): Maggots wander away to pupate after bowel evacuation. **(F)** Transition between advanced decay and dry remains stages (Day 30): Due to precipitation on several days, colder temperatures, and thus reduced insect activity, decomposition has slowed down. **(G)** Dry remains (Day 41): skeletal remains were displaced by a red fox (Vulpes vulpes).

Morphologically distinct decomposition stages were defined as *fresh*,* putrefaction*,* bloated* (interpreted as a visible external sign of putrefaction; anaerobic decomposition), *post-bloated* (active decay), *advanced decay*, and *dry remains* (aerobic decomposition), based on definitions provided by^55^ and Lee Goff (2009) (Table 1; Fig. 2).

**Table 1:** The six morphologically distinguishable decomposition stages used in the statistical analysis, based on Anderson and Van Laerhoven (1996) and Lee Goff (2009).

Decomposition stage	Distinguishing features
***1 - Fresh***	First post-mortem stage, cooling of the body temperature after death and chemical breakdown (autolysis) of the body
***2 - Putrefaction***	Digestion of body tissue by anaerobic bacteria
***3 - Bloated***	Visible inflation of the abdomen due to microbial gas production
***4 - Post-bloated***	Active decay; skin breakage due to gas accumulation and insect or maggot activity, deflation of the body, removal of most of the body’s flesh by large maggot masses
***5 - Advanced decay***	Carcass left by the maggots for pupation, continued removal of the remaining flesh by Coleoptera and other arthropods
***6 - Dry remains***	Carcass skeletonized; only bones and skin with fur remaining

Environmental variables included *habitat* (*open* or *closed)*,* soil condition* (*wet* or *dry)*, based on our habitat assessment, and *air temperature*, extracted from the TOMST weather stations at each carcass site and calculated as the daily mean, to account for possible influences on the decomposition process^56^. *Series ID* was considered a grouping variable, indicating simultaneous carcass placements within 16 series.

The assessed carcass-related factors included *carcass temperature* (daily mean carcass temperature in °C), *carcass condition* (*previously frozen* or *not frozen)*, and *pH in the carcass*, calculated as the mean pH per day (-log_10_[mean a(H^+^)]).

### Statistical analysis

#### Carcass temperature

We analysed the effect of several predictor variables on the dependent variable *carcass temperature* using a generalized linear mixed effects model (GLMM) (Table 2 - Model 1). The predictor variables were *decomposition stage* (included as a categorical variable), *air temperature*, *habitat*, *soil condition*, and *carcass condition*. The effect of *air temperature* was included in the simplest non-linear regression model by using a second-order raw polynomial. We included an interaction between *decomposition stage* and *air temperature* (polynomial) to assess their combined effect on *carcass temperature*, because air temperatures drives carcass decomposition^57^. The *series ID* was included as a random intercept, and *carcass ID* to account for the nested structure of the carcass sites belonging to the respective placement series.

**Table 2:** Characteristics of the GLMM-models and variables used (X) in the statistical analysis.

	Parameters/units	Model 1 Carcass temperature	Model 2 pH in carcass muscle	Model 3 pH in carcass rectum	Model 4 Soil pH
**Response variables**					
**Carcass temperature**	Daily average	X			
**Muscle pH**	Daily mean of H^+^-activity		X		
**Rectal pH**	Daily mean of H^+^-activity			X	
**Soil pH**	Daily mean of H^+^-activity				X
**Predictor variables**					
**Decomposition stage**	Fresh, putrefaction, bloated, post-bloated, advanced decay, dry remains	X	X	X	X
**Air temperature**	Daily average	X			
**Carcass temperature**	Daily average		X	X	
**Habitat type**	Open, closed	X			
**Soil condition**	Wet, dry	X			X
**Carcass condition**	Frozen and thawed, freshly shot and not frozen	X			X
**Model structure**					
**Family**		Gaussian	Gamma (log-link)	Gamma (log-link)	Gamma (log-link)
**Random effect**		Series ID/Carcass ID	Carcass ID	Carcass ID	Series ID/Carcass ID

### pH in the carcasses

We first tested for the difference in muscle and rectal pH measurements using a Wilcoxon test. We then fitted two GLMMs with a gamma distribution and a log-link function to test the effect of *decomposition stage* and *carcass temperature* on *pH dynamics* measured in *muscle* tissue (Table 2 - Model 2) and in the *rectum* (Table 2 – Model 3). *Decomposition stage* and *carcass temperature* were included as an interaction term, as carcass temperature drives microbial (bacteria and fungi) decomposition, and a rise in temperature is caused by the activity of maggots^37,58^. *Carcass ID* was included as a random effect.

### Soil pH

Lastly, a GLMM with a gamma distribution and a log-link function was used to determine which parameters, such as *decomposition stage*,* soil condition*, and *carcass condition*, influenced the pH dynamic in the topsoil underneath the carcasses (Table 2 - Model 4). Similar to Model 1, *series ID* and *carcass ID* were included as a nested random effect.

Correlations between predictor variables were examined a priori to assess potential relationships and multicollinearity using a Spearman rank correlation coefficient, visualized using the ‘corrplot’ package^59^. Collinearity was determined to be < 0.7^60^. The GLMMs were calculated using the R package glmmTMB^61^ and validated with residual diagnostics using the DHARMa package^62^. Model predictions were generated using the ggeffects package and the argument “marginalmeans”, i.e., non-focal predictors were set to their mean (numeric variables) or marginalized over the levels or “values” for factors and character vectors^63^. All statistical analyses were performed using R 4.2.2^64^. Statistical significance was assumed at *P* ≤ 0.05.

## Results

### Carcass temperature

**Fig. 3:**
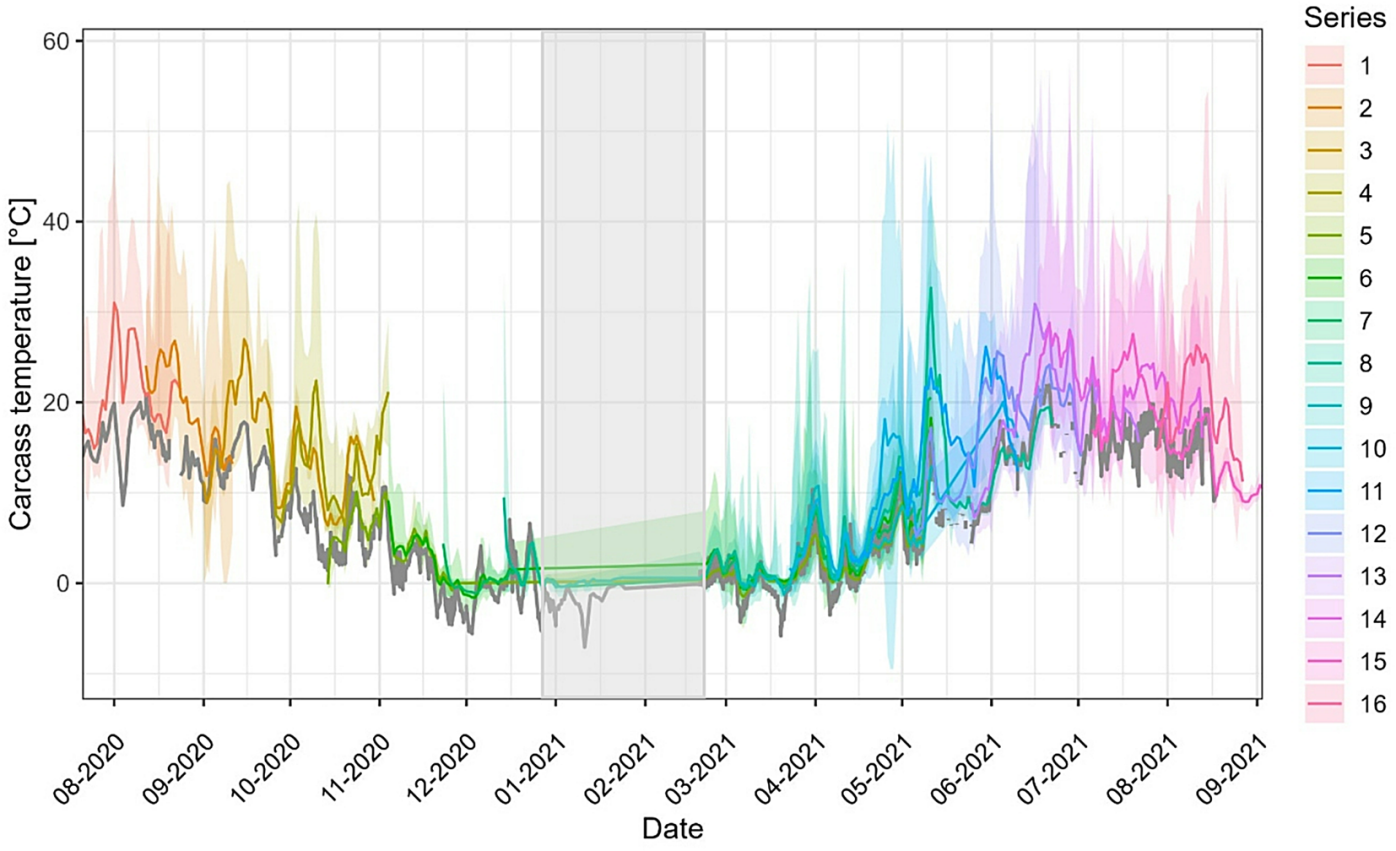
Average daily carcass temperatures for each placement series, based on data of the four respective wild boar carcasses. Carcass placement was suspended in January and February 2021, temperature measurements were therefore limited during that period (grey rectangle). The grey line represents the daily mean air temperature. Coloured lines represent the carcass temperatures for 16 different placement series, and shaded areas the respective 95% confidence intervals.

**Fig. 4:**
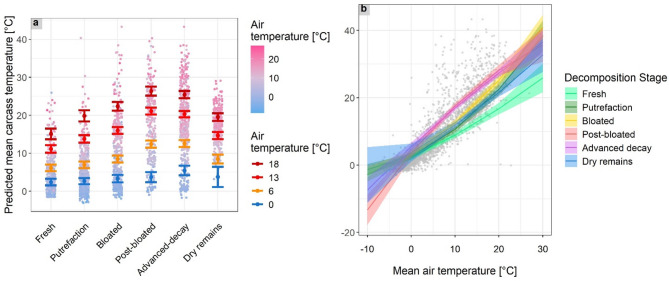
**a**) Predicted mean carcass temperatures (°C) and confidence intervals for each wild boar carcass decomposition stage as a function of air temperature. The figure illustrates the interaction effect between decomposition stage and air temperature. The increase in carcass temperature was faster and higher with increasing air temperature, with the highest carcass temperatures estimated during the post-bloated and advanced decay stages. Carcass temperatures were predicted at the first quartile (0 °C, blue), mean (6 °C, yellow), third quartile (13 °C, orange-red), and 95th percentile (18 °C, dark-red) of the measured and averaged daily air temperatures. Observed carcass temperatures are depicted as dots, coloured with the related air temperature (°C), ranging from colder (blue) to warmer (pink) temperatures. **b**) Predicted changes in the mean daily carcass temperature (°C) in relation to the mean daily air temperature (°C), depending on the decomposition stage. Grey points represent the observed carcass temperatures.

The recorded average daily carcass temperatures ranged from − 3.1 to 43.3 °C, with a median of 7.5 °C and a mean of 9.8 °C. The minimum and maximum single recordings were − 9.5 and 58.0 °C, respectively (for recorded carcass temperature in each decomposition stage, see Fig. 4a, displayed as dots). Carcass temperatures varied over the year, with higher values occurring during warmer months (Fig. 3).

In seven carcasses, temperatures at time exceeded 50 °C. Four events above that threshold (consecutive measurements) lasted 1 h, and six events over 1.5 h, with a maximum duration of 5.5 h. Two carcasses reached temperatures above 55 °C, with one event lasting 2 h. Detailed information on the number of events and the duration above specific carcass temperatures is provided in the Supplementary Table S2.

Model 1 showed that the carcass temperature increased during decomposition (Supplementary Table S3), with the highest temperatures occurring during the aerobic decay stages, including stage 4 - post-bloating and 5 - advanced decay (predicted average + SE = 12.6 ± 0.48 and 12.7 °C ± 0.46 °C, respectively). As decomposition progressed, the temperature decreased until stage 6 - dry remains was reached. Similarly, the interaction between *decomposition stage* and *air temperature* had the highest effect size during the aerobic decay stages (decomposition stages 4 and 5, Supplementary Table S3), with more pronounced effects at elevated air temperatures, indicating a significant combined effect during those stages (Supplementary Table S3, Fig. 4a). With rising air temperature, carcass temperature increased, following a linear distribution with a quadratic trend (Supplementary Table S3, Fig. 4b). Carcass temperatures were higher in habitats with an open than with a closed canopy (predicted average + SE for closed and open habitats = 8.62 ± 0.46 °C and 9.92 ± 0.45 °C, respectively; Supplementary Table S3; Figure S3). Neither the placement of a carcass on wet vs. dry soil nor the carcass condition (not frozen/frozen and thawed) had a significant effect on carcass temperature.

### pH in the carcasses

The average daily pH values in the carcasses ranged from 4.7 to 9.5 (median = 6.4, mean = 6.7). The minimum recorded pH in a single measurement was 4.5 (muscle; rectum = 5.1), and the maximum single pH was 9.7 (rectum, muscle = 9.6). pH values < 5 lasted for more than 3 and 5 days for two carcasses in the putrefaction and bloated decomposition stages, respectively.

There was a significant difference in the pH between measurements methods (Wilcoxon test: *P* < 0.01, Supplementary Figure S4), with higher pH values measured rectally. The average daily pH in the hind thigh muscle of eight wild boar carcasses ranged from 4.7 to 9.5, with a median of 6.3 and a mean of 6.7. Rectal pH measurements ranged from 5.1 to 9.5, with a median of 6.6 and a mean of 6.9. An overview of the muscle and rectal pH levels measured during the different carcass decomposition stages is provided in Table 3.

**Table 3:** Summary statistics of daily average, median, minimum, maximum, and quartile pH for each decomposition stage. Measurements were obtained in situ in wild Boar carcasses with pH-probes inserted in the Hind thigh muscle and rectally.

Decomposition stage	Average pH muscle/rectal	Median pH muscle/rectal	Min pH muscle/rectal	Q25 pH muscle/rectal	Q75 pH muscle/rectal	Max pH muscle/rectal
Fresh	5.8/6.0	5.7/6.0	4.8/4.0	5.63/5.85	5.83/6.28	7.7/6.6
Putrefaction	5.8/6.3	5.8/6.4	4.2/5.5	5.44/6.05	5.95/6.65	7.9/8.8
Bloated	5.7/5.7	5.5/5.6	4.9/5.0	5.28/5.36	5.71/5.97	7.7/9.5
Post-bloated	6.7/6.7	6.6/6.5	5.2/5.5	5.70/6.09	7.45/6.78	9.6/9.1
Advanced decay	7.7/8.1	7.9/8.3	5.6/6.3	6.96/7.85	8.52/8.57	9.1/9.7
Dry remains	8.6/7.5	8.6/7.4	7.1/6.6	8.26/6.75	9.16/7.65	9.5/9.0

**Fig. 5:**
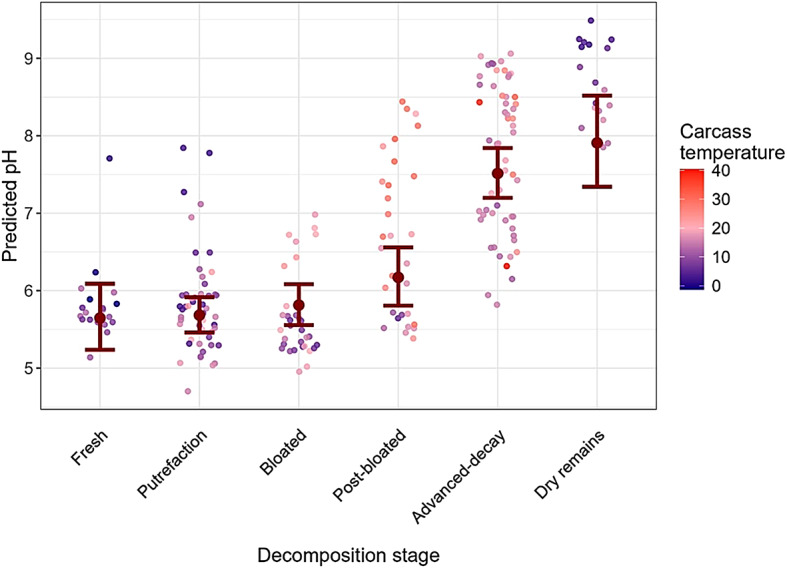
Predicted pH and confidence intervals based on measurements in the hind thigh muscle of decomposing wild boar carcasses. Points represent the observed values and are coloured in the corresponding carcass temperatures (colder temperatures in blue, warmer values in red). Temperatures were highest during the post-bloated and advanced decay stages.

Model 2, which tested the effects of carcass temperature and decomposition stage on carcass pH in muscle, showed significant influences of the decomposition stage and the interaction with carcass temperature. The pH increased beginning at stage 3 - bloated and reached the highest values at the end of the decomposition (predicted average + SE = 7.91 ± 0.31; Fig. 5). The interaction effect of carcass pH with carcass temperature was particularly notable at stages 3 - bloated, 4 - post-bloated, and 5 - advanced decay of decomposition, with the strongest effect for stage 4 (Supplementary Table S4; Figure S5).

Model 3 tested the effect of the decomposition stage and carcass temperature on rectal pH. The analysis revealed an increase in pH during the aerobic decay stages (stages 4 - post-bloated and 5 - advanced decay), with the highest values reached during the advanced decay stage (predicted average + SE = 7.96 ± 0.18; Supplementary Figure S6). The positive interaction effect with carcass temperature was particularly notable at decomposition stages 4 - post-bloated, and 5 - advanced decay (strongest positive effect for stage 4), while stage 2 - putrefaction was characterized by a negative interaction effect (Supplementary Table S5).

### Soil pH

**Fig. 6:**
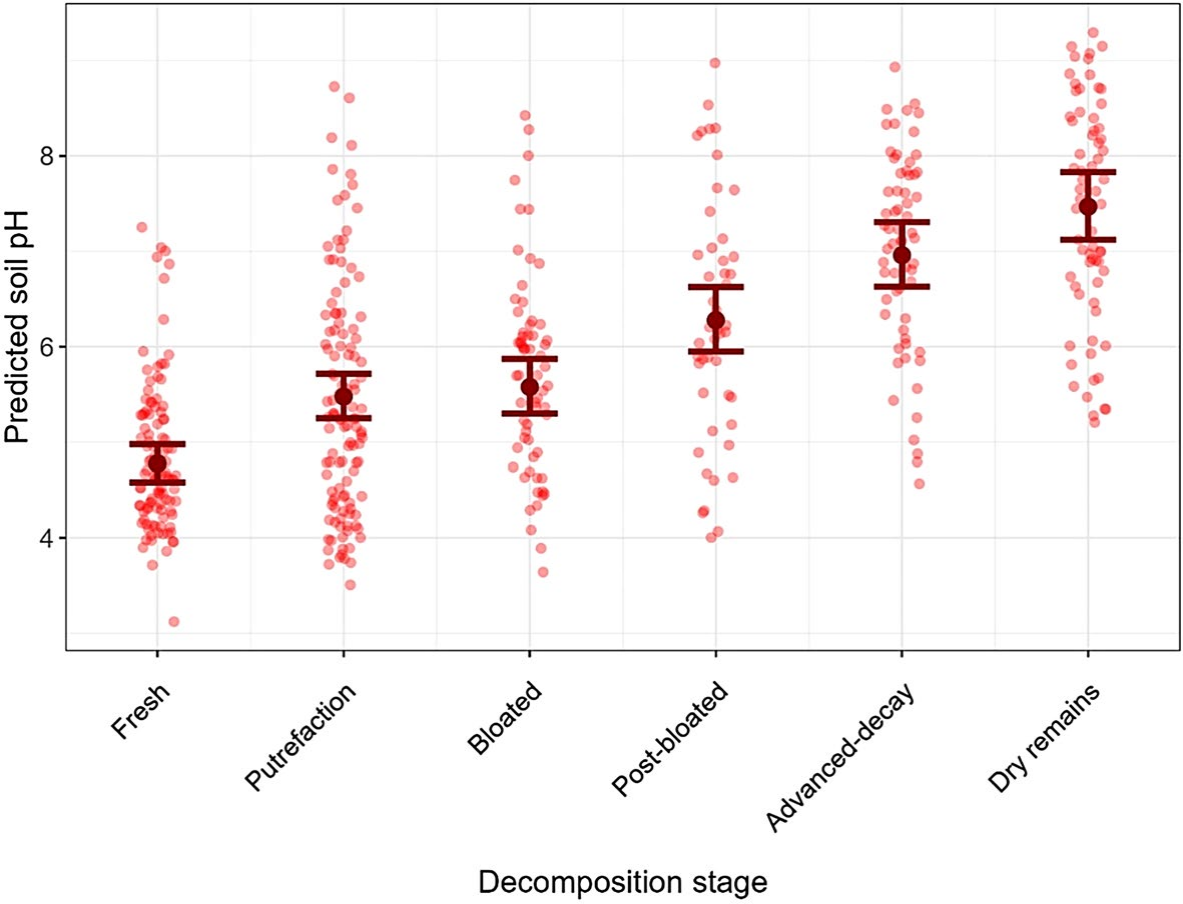
Predicted pH with confidence intervals for the topsoil underneath wild boar carcasses during decomposition. Predictions are based on “marginalmeans”. Red dots depict observed soil pH at each decomposition stage.

The pH of the topsoil under the carcasses ranged from 3.4 to 9.1 (median = 5.8, mean = 6.0). Two carcasses revealed pH values below 4 for measurements until day 23 and day 30, when the respective carcasses, placed in closed habitats and on dry soil, were in the putrefaction stage. Model 4, which tested the effects of decomposition stage as well as soil and carcass condition on soil pH underneath the carcasses, revealed a significant increase in pH for all decomposition stages compared to the first stage (strongest effect for the dry remains stage: predicted average + SE = 7.47 ± 0.18; Supplementary Table S6, Fig. 6). Soil moisture and carcass condition (not frozen vs. previously frozen and thawed) did not affect the soil pH.

## Discussion

This study explored the dynamics of temperature and pH in decomposing wild boar carcasses to evaluate their potential for pathogen inactivation. We found that carcass temperatures and pH values varied widely over the decomposition process, with temperature peaking during the post-bloating and advanced decay stages (max. 58 °C). Air temperature and open habitats were important drivers of carcass temperature. Carcass pH and the pH of the underlying topsoil ranged from 4.5 to 9.7, and from 3.4 to 9.1, respectively, with both increasing significantly over the course of the decomposition.

### Carcass temperature

We observed a consistent increase in carcass temperature during the early (putrefaction and bloated) and aerobic stages of decomposition (post-bloated and advanced decay). Although the initial rise, during anaerobic decomposition, is attributed to microbial activity, the primary driver for the subsequent increase in temperature, during aerobic decomposition, was likely the metabolic activity of maggots^65,66^ which require ambient temperatures above 10 °C^58,67^. Maggots accelerate the decomposition process, leading to rapid skeletonization in summer, whereas the activity of decomposers is limited during colder temperatures, leading to lower carcass temperatures (Fig. 4a)^41^. These seasonal differences account for the occurrence of skeletonization within days in summer but carcass persistence over several months during cold winters with frost^56,57^ (Supplementary Figure S2). Carcasses placed in winter were often in active decay simultaneously with those placed in spring, as also observed by^68^. Our findings align with previous studies reporting a positive relationship between carcass temperature and ambient air temperature^69^. Amendt et al. (2017) and^70^ measured temperatures in domestic pig carcasses in May and August and found that they peaked around 40 °C. Similar to our study^71^, investigated the temperature changes in wild boar carcasses measured rectally in August and September. They reported temperatures 10–20 °C higher than the ambient air temperature, likely as the warm ambient temperatures enhanced maggot activity. Seven carcasses in our study reached temperatures above 50 °C, with 11 events lasting 60 min or longer. Those carcasses had been placed in open habitats (solar heating) and the events mostly occurred during advanced decay when air temperatures reached around 33 °C. It should also be noted that, as the decomposition of muscle tissues and organs nears completion, temperature probes may be exposed. Consequently, late-stage measurements might be more strongly influenced by air temperature and solar heating than measurements made during earlier decomposition stages. In additions, carcass temperatures in different body parts may vary. In the study by^72^temperatures under the chest were higher than rectal temperatures. The latter may fluctuate as gut contents are expelled during decomposition due to the rising internal pressure from metabolically produced gases, with maggots then moving through natural body entrances and may raising the temperature at their sites of passage^72^. Body structures that do not contain extensive musculature as a protein source for fly larvae are likely to be infested less often or by fewer maggots, such that temperatures in those carcass parts might not be as high as in others (see Supplementary Figure S7, for a thermal image of a decomposing wild boar).

The thermal sensitivity of pathogens varies widely such that the internal carcass temperature and its duration affect pathogen viability. Pathogens such as CSFV, *E. coli*, Salmonellae, and *Mycobacterium bovis* are inactivated within an hour at temperatures > 50 °C^73,74^ whereas the bacterium *Brucella abortus* and hepatitis E virus require temperatures > 60 °C for their inactivation^39,73^. By contrast, avian influenza viruses are inactivated after 15 min at temperatures as low as 40 °C^75^. ASFV is effectively inactivated within an hour by temperatures > 53 °C^14,40^. Prolonged exposure of infected tissue is required at colder temperatures; for example, ASFV in pig bones stored at room temperature was not infectious after a week but at 4 °C it remained infectious for up to one month. Skin tissue remained infectious for 6 months at 4 °C and for 3 months at room temperature, while infectious virus in muscle was detected for up to 3 months at 37 °C^16^. Infectivity in well-perfused organs such as the spleen, kidneys, and lungs was reduced by 90% (D-values) after 19, 6, and 21 days at 4 °C, and 2, 3, and 1 days at 23 °C, respectively^76^.

Comparisons of these temperature thresholds required for inactivation within a short duration of time with our measurements (Supplementary Table S2) suggest that the inactivation of pathogens such as CSFV, ASFV or Salmonellae in wild boar carcasses may be possible (at 50 °C within 1 h), but only in a relative small proportion of the cases (in our study, 7 out of 74 carcasses) and likely only during the aerobic stages of decomposition (post-bloating and advanced decay) in the warm season and in open habitats. Hepatitis E virus, however, would likely remain active. Further research is needed to explore pathogen survival in carcasses with prolonged exposure at lower temperatures. Mazur-Panasiuk et al. (2020) suggested a low stability of ASFV at temperatures in the range of 20–25 °C, depending on the organ and fomite type, such that inactivation might also be possible at lower temperatures. Comparable temperature patterns during the decomposition of carcasses of species similar in size to wild boar can be expected, but heat development in smaller carcasses might differ. Although pathogen inactivation due to heat sensitivity will only be possible during warmer seasons, as carcasses do not reach sufficient temperatures for inactivation during colder seasons, higher air and carcass temperatures, also facilitated by solar radiation, can lead to carcass dehydration and thus to pathogen inactivation. Nonetheless, since higher temperatures are mostly reached during active decay, we believe that the risk of disease transmission to other animals, scavengers, or, in the case of ASF, to wild boar, will be particularly high during the fresh to bloated stages.

### Carcass pH

While our results showed a continuous increase in carcass pH during decomposition, in previous studies pH values underwent an initial decline shortly after the animal’s death^48,69,77^. The decrease can be attributed to autolysis, which reduces blood pH as the decomposition products accumulate. Within cells, anaerobic fermentation of lactic acid from muscle glycogen further lowers the pH, leading to cell structure breakdown^78,79^. The absence of an initial pH decline in the wild boar carcasses likely reflects the fact that our reliance on purely morphological assessments did not allow autolysis to be distinguished from putrefaction. Moreover, since our study mostly made use of carcasses that were frozen shortly after the animal had died, the initial decline in pH may have been missed. However, some individual measurements showed a pH drop to as low as 4 at the beginning of decomposition.

With the progression of decomposition, the accompanying rise in carcass temperature led to an increase in pH, which was particularly notable as aerobic decomposition progressed (post-bloated and advanced decay). Body cell degradation is followed by putrefaction, wherein microbes ferment proteins, carbohydrates, and lipids while further producing fatty acids, amino acids, and dissolved inorganic nitrogen^41,44,80^ such that the internal environment of the carcass becomes increasingly alkaline. Junkins et al. (2019) measured the pH in maggot masses and found an increase from 6.4 to 7.7 up to 128 h post-mortem^49^.

However, accurately measuring the pH of carcass tissue is challenging, as pH is usually measured in liquids rather than solid media. The pH probe used in our study was designed for soft and solid media and its accuracy was validated using handheld pH indicator strips. To the best of our knowledge, only Hunnam et al. (2018) also assessed the pH in naturally decomposing carcasses, but only by taking hourly hand-held measurements in various carcass parts, including muscle, within the first 26 h post-mortem. Neither the pH meter that was used nor their methodology for in situ pH measurement was specified. Nonetheless, their measurements, taken during the initial 24 h after carcass placement (pH 5.3 to 6.5), were similar to our own. The slightly higher pH readings in the rectum than in muscle tissue in our study may have been due to the influence of the intestinal content. However, the differences were minor, with mean pH values of 6.7 and 6.9 for muscle and rectal measurements, respectively. In the study by Hunnam et al. (2018), pH measurement at different anatomic locations also differed, by up to 0.5 units.

### Soil pH

Soil pH continuously increased during the decomposition process, particularly with the onset of aerobic decomposition, when body fluids enter the soil. Similar observations were reported in studies of the soil underlying the carcasses of pig, rabbit, pika, beaver, mice, and humans^42–44,81–84^. For example, the soil pH under decomposing pig carcasses increased from 6.4 up to 7.4^81,82^. MacDonald et al. (2014) documented an increase in the pH of the soil beneath 18 kangaroo carcasses, lasting at least 24 weeks including the more advanced dry remains stage. This increase was attributed to the input of alkaline putrefaction products and the enhanced oxidative microbial decomposition of carbohydrates and amino acids, which resulted in oxygen-depleted topsoil beneath and surrounding the remains^44,80,85^. Anaerobic conditions prevent the oxidation of ammonia (NH_3_) and ammonium (NH_4_^+^) by nitrifying bacteria and the production of saltpetre acid, which would lead to a drop in soil pH. Long-term studies below three pig carcasses revealed a rise in pH for up to 200 days, followed by a gradual decline to nearly baseline levels after 760 days^86^. In extreme cases, the pH of the soil below large-sized carcasses of bison (*Bison bonasus*) remained elevated for up to 6 years^87^. However, scavengers can scatter a carcass several times^88^ restricting the effects of carcass decomposition on soil pH at the site of the animal’s death, similar to an altered nutrient leakage from scavenged carcasses (Wenting et al. 2024).

Notably, the soil in our study area consists mainly of slightly acidic cambisols. An unpublished analysis of BFNP topsoil during 1990–2016 determined a mean pH of 3.87 (0–5 cm depth, *n* = 20) and 3.55 (0–10 cm depth, *n* = 309) without significant changes over time. This is in accordance with the unaltered pH in the mineral topsoil of other German forests^89^. Additionally, the prevailing ion-exchange aluminium/aluminium-iron buffer system in these soils prevents the pH from dropping far below 3.5^90^. Therefore, investigations of the influence of soil pH on virus capsid stability in disease management need to take into account the local soil type, as different soil types might influence the pH changes during carcass decomposition^91^. Seasonal differences must also be considered, given that alterations in soil pH may be less obvious in winter^92^ due to the delayed decomposition at colder temperatures^57^.

The condition of the exposed carcass could also influence the pH changes in soil. Our study used both previously frozen as well as freshly shot, not frozen wild boar. Freezing and thawing (which naturally also occur in winter and early spring) can affect bacterial metabolism and tissue conditions, and therefore tissue liquefaction rates^68^ with possible effects on pH. However, there were no significant differences in the temperature or pH changes between frozen and not frozen wild boar carcasses, as their decomposition rate also did not differ significantly^57^.

Pathogens vary in their pH sensitivity. For instance, *Mycobacterium tuberculosis* var. *bovis* maintains activity at a pH as low as 4–5 and is capable of prolonged survival in the soil (Allen, Ford, and Skuce 2021). *Brucella microti* withstands extreme acidity (pH 2.5), whereas *Brucella suis* survives only above pH 4.5^93^. CSFV remains infectious at pH 8–9 but is rapidly inactivated below pH 4^94^. Some pathogens, including foot-and-mouth disease virus and avian influenza virus, are more sensitive to alkaline conditions; both of these pathogens lose their infectivity below slightly acidic conditions, around pH 6.5^69,95^. ASFV, by contrast, tolerates a broader pH range and remains infectious between pH 4 and 9^14,50^. However, only one study has investigated the pH changes in carcasses in the context of wildlife diseases. In their study of foot-and-mouth disease virus, Hunman et al. (2018) found that viral activity in pig carcasses decreased within 16 h post-mortem, as the pH dropped below 6. In the present study, the pH range measured in the wild boar carcasses and surrounding soil was between 4 and 9, which is within the tolerance of the wildlife pathogens noted above, with the exception of avian influenza virus. A study examining the stability of ASFV in different soil types detected active viruses in yard soil (pH 6.7) for up to one week at 25 °C, but not in forest soil (pH 4.1). In swamp mud (pH 5.1), infectious ASFV persisted only 3 days^24^. In soil from cadaver decomposition islands [CDI, highly concentrated island of fertility^96^ (pH = 3.2), active ASFV was detected for up to one week^16^. We measured in only two carcasses soil pH values below 4 up to day 23 and day 30, when the carcasses, in closed habitats and dry soil, were in the putrefaction stage. Mazur-Panasiuk et al. (2020) examined ASFV survival times in different organic layers. The authors placed infected spleen on leaf litter, straw, hay, and grain and determined a 90% loss of virus infectivity (D-values) at a constant temperature of 4 °C after 3, 17, 21, and 13 days, respectively, but no infectious virus at 23 °C after 7 days on all matrices. These results highlight the potential impact of soil pH on pathogen inactivation, considering additional influencing factors such as soil type and temperature^97^. Furthermore, with emerging ASFV variants^98^ alterations in resistance to pH and temperature might be possible.

Although the carcass temperatures and pH levels measured in this study align with conditions allowing ASFV persistence, interactions with other environmental factors may lead to virus inactivation. It should be considered that environmental conditions, such as sunlight acting as a natural disinfectant^16^ and different vegetation surfaces, can influence the tenacity of certain pathogens, as demonstrated for ASFV^76^. Additionally, our results might only be valid for carcasses similar in size to juvenile and adult wild boar and for the local soil type.

## Conclusions

This study investigated the post-mortem temperature and internal pH changes throughout the decomposition process of wild boar carcasses. From an initially measured, low pH value of 4.5, the pH of the carcasses increased during aerobic decomposition, accompanied by a continuous rise in soil pH. Elevated temperatures potentially conducive to virus inactivation primarily occurred during the advanced decay stages of the carcasses and only in summer. Our results suggest that the changes in temperature and pH during the natural decomposition of wild boar carcass may inactivate some, but not all wildlife pathogens, but further research is needed to study the effect of specific environmental factors on the tenacity of different pathogens.

## Electronic supplementary material

Below is the link to the electronic supplementary material.

Supplementary Material 1

Supplementary Material 2

## Data Availability

The datasets generated and/or analysed during the current study are available in the DRYAD repository, [https://datadryad.org/stash/share/ZvJhQDnqEQW92GmQ6VuZcutBjSWx-evpGC73vZHpb_o].
