# Supplementary material for: Temperature and pH dynamics during carcass decomposition and implications for disease management
Source: Sci Rep. 2025 Jul 16;15:25795. doi: 10.1038/s41598-025-07716-w (PMC12267392; doi:10.1038/s41598-025-07716-w)
Supplement: Supplementary file 1 — Supplementary Material 1 [file 41598_2025_7716_MOESM1_ESM.pdf]

Supplementary material

Temperature and pH dynamics during carcass decomposition and implications for disease management

Janine Rietz, Burkhard Beudert, Nicolas Ferry, Lukas Böcker, Franz J. Conraths, Carolina Probst, Andreas Zedrosser, Helmut Küchenhoff, Martin Hais, Jens Schlüter, Tomas Lackner, Christian von Hoermann, Jörg Müller, Marco Heurich

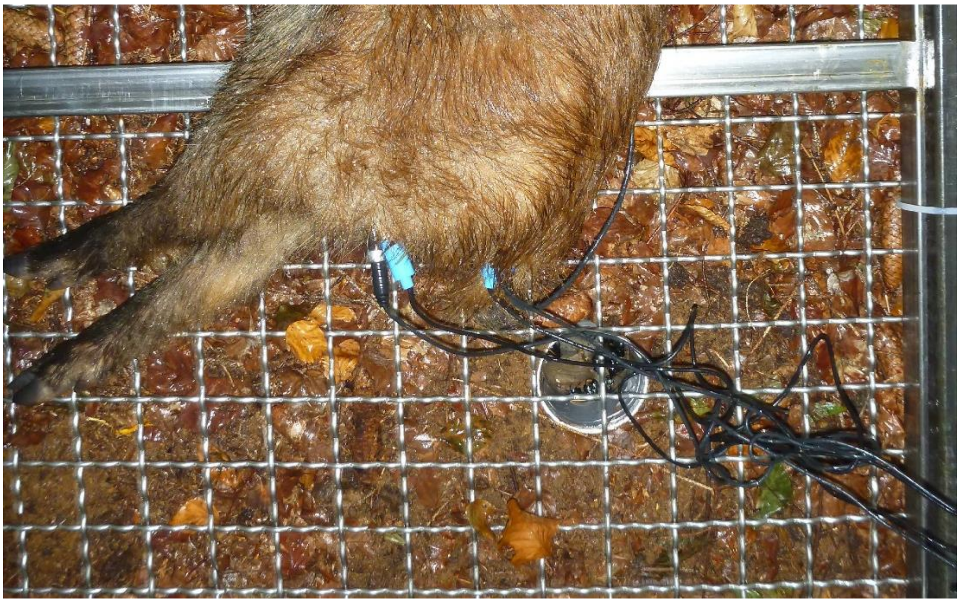

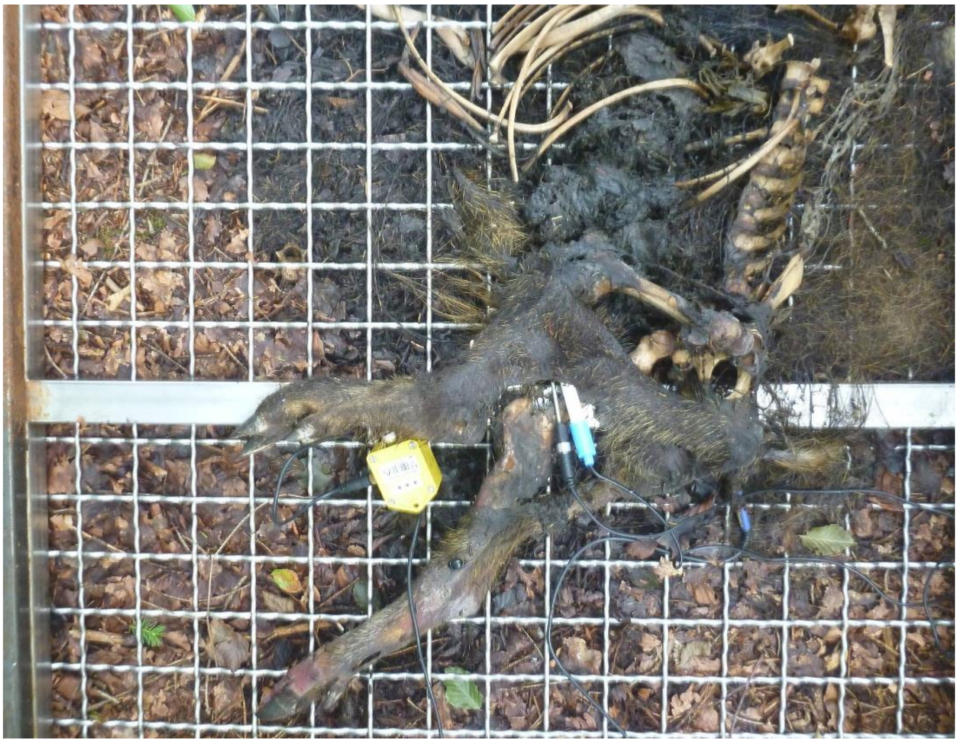

Figure S1: pH and temperature probes inserted rectally and into the hind thigh muscle of two wild boar carcasses placed in cages. The upper photo shows a carcass shortly after placement, with initial pH and temperature measurements. The lower photo shows a carcass at the onset of skeletonization (beginning of the dry remains stage), marking the end of the field experiment. The pH and temperature probes, previously positioned between the skin and bones, were removed shortly thereafter.

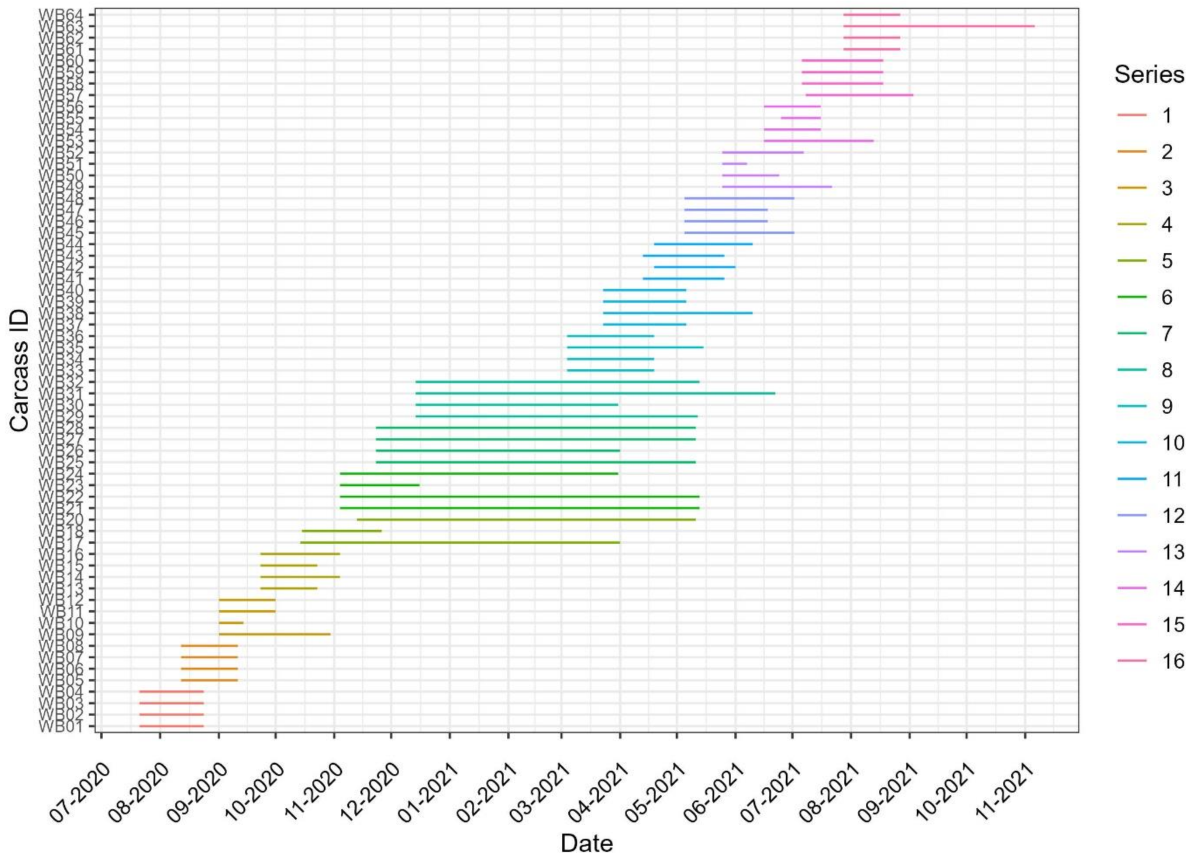

Figure S2: Time span of carcass monitoring and measurements for each carcass, grouped by placement series.

Table S1: Overview of the environmental and carcass-related variables as well as the number of sampling days for each carcass/plot ID. Plot ID labels: WB = wild boar, CC = carcass in cage. Fresh refers to freshly shot and not frozen (yes) or frozen (no) carcasses. Sampling days indicate the number of soil samples taken.

**Series ID**	**Date**	**Plot ID**	**Fresh**	**Sex**	**Weight (kg)**	**Habitat**	**Soil**	**Sampling days**
1	21.07.2020	WB01	yes	male	28.8	open	wet	10
1	21.07.2020	WB02	no	female	41.0	open	dry	10
1	21.07.2020	WB03	no	female	28.0	closed	wet	10
1	21.07.2020	WB04	no	female	29.5	closed	dry	10
1	21.07.2020	CC01	no	male	33.5	closed	dry	9
2	12.08.2020	WB05	no	male	36.0	open	dry	9
2	12.08.2020	WB06	yes	female	13.6	closed	dry	3
2	12.08.2020	WB07	no	female	45.5	open	wet	3
2	12.08.2020	WB08	no	female	9.4	closed	wet	3
2	12.08.2020	CC02	no	male	30.0	closed	dry	8
3	01.09.2020	WB09	no	female	74.0	closed	dry	4
3	01.09.2020	WB10	no	male	10.0	open	wet	9
3	01.09.2020	WB11	yes	female	9.0	closed	wet	4
3	01.09.2020	WB12	no	male	49.5	open	dry	4
3	01.09.2020	CC03	no	male	21.5	closed	dry	9
4	23.09.2020	WB13	no	male	16.0	open	wet	4
4	23.09.2020	WB14	no	female	50.0	closed	wet	13
4	23.09.2020	WB15	yes	male	19.3	closed	dry	4
4	23.09.2020	WB16	no	female	38.0	open	dry	4
4	23.09.2020	CC04	no	male	24.5	closed	dry	9
5	13.10.2020	WB17	no	female	48.0	closed	dry	17
5	13.10.2020	WB18	yes	male	25.5	open	wet	5
5	13.10.2020	WB19	no	male	20.0	open	dry	5
5	13.10.2020	WB20	no	female	22.0	closed	wet	8
5	13.10.2020	CC05	no	male	49.0	closed	dry	14
6	04.11.2020	WB21	no	female	21.6	open	wet	8
6	04.11.2020	WB22	yes	female	29.7	closed	dry	8
6	04.11.2020	WB23	no	female	76.0	closed	wet	7
6	04.11.2020	WB24	no	male	11.5	open	dry	6
6	04.11.2020	CC06	no	male	43.0	closed	dry	9
7	23.11.2020	WB25	no	female	37.0	open	wet	10
7	23.11.2020	WB26	no	male	59.0	closed	dry	6
7	23.11.2020	WB27	no	female	73.0	open	dry	9
7	23.11.2020	WB28	no	female	25.5	closed	wet	10
8	14.12.2020	WB29	yes	male	62.0	closed	wet	4
8	14.12.2020	WB30	no	female	38.5	open	dry	8
8	14.12.2020	WB31	no	male	75.0	closed	dry	7
8	14.12.2020	WB32	no	male	58.0	open	wet	4
9	03.03.2021	WB33	no	male	37.0	open	dry	15
9	03.03.2021	WB34	yes	male	43.0	closed	dry	15
9	03.03.2021	WB35	no	male	58.3	closed	wet	15
9	03.03.2021	WB36	no	female	32.5	open	wet	7

Series ID	Date	Plot ID	Fresh	Sex	Weight (kg)	Habitat	Soil	Sampling days
10	23.03.2021	WB37	no	female	50.0	closed	wet	13
10	23.03.2021	WB38	no	female	68.0	closed	dry	14
10	23.03.2021	WB39	no	female	41.8	open	wet	5
10	23.03.2021	WB40	yes	male	33.8	open	dry	6
11	13.04.2021	WB41	yes	male	58.5	open	wet	5
11	13.04.2021	WB42	no	female	74.0	closed	dry	5
11	13.04.2021	WB43	no	male	54.0	open	dry	5
11	13.04.2021	WB44	no	male	60.0	closed	wet	11
11	13.04.2021	CC07	no	female	60.0	closed	dry	11
12	05.05.2021	WB45	yes	male	57.5	open	dry	11
12	05.05.2021	WB46	no	female	54.0	closed	wet	8
12	05.05.2021	WB47	no	female	45.0	open	wet	5
12	05.05.2021	WB48	no	male	44.5	closed	dry	8
13	25.05.2021	WB49	no	male	62.0	open	dry	4
13	25.05.2021	WB50	no	female	58.0	closed	dry	4
13	25.05.2021	WB51	no	male	45.7	closed	wet	2
13	25.05.2021	WB52	yes	male	56.0	open	wet	10
14	16.06.2021	WB53	no	female	44.0	open	wet	4
14	16.06.2021	WB54	no	male	55.5	open	dry	5
14	16.06.2021	WB55	yes	male	93.0	closed	wet	4
14	16.06.2021	WB56	no	male	50.0	closed	dry	9
14	16.06.2021	CC08	no	male	48.0	closed	dry	5
15	06.07.2021	WB57	yes	male	57.0	closed	wet	10
15	06.07.2021	WB58	no	male	40.2	closed	dry	4
15	06.07.2021	WB59	no	female	35.0	open	dry	4
15	06.07.2021	WB60	no	male	56.0	open	wet	4
15	06.07.2021	CC09	no	male	29.6	closed	dry	8
16	27.07.2021	WB61	no	male	45.0	closed	wet	4
16	27.07.2021	WB62	no	female	54.0	open	dry	10
16	27.07.2021	WB63	yes	male	95.0	closed	dry	4
16	27.07.2021	WB64	no	male	53.0	open	wet	4
16	27.07.2021	CC10	no	male	50.0	closed	dry	9

Table S2: Number of carcasses and number of events (nEvents) above specific carcass temperature thresholds (30–55°C) lasting 30, 60, 90 min, and > 90 min. The median, mean, and maximum durations (in minutes) of these events are also reported. Due to sampling frequency, the minimum duration was consistently 30 min. Temperatures > 45°C were reached only during aerobic decomposition (stages 4 - post-bloated, 5 - advanced decay, and 6 - dry remains), while temperatures between 35 and 45°C were also reached during stage 2 -putrefaction and stage 3 - bloated.

Carcass temperature (°C)	Number of carcasses	nEvents 30 min	nEvents 60 min	nEvents 90 min	nEvents > 90 min	Median (min)	Mean (min)	Maximum (min)
**55**	2	2	0	1	1	60	68	120
**50**	7	2	4	1	6	90	129	330
**45**	12	10	8	7	18	90	112	300
**40**	23	40	18	10	62	90	176	2700
**35**	31	57	34	24	141	150	312	4530
**30**	41	72	50	23	236	210	451	7020

Table S3: Parameter estimates of generalized linear Model 1, evaluating the effect of decomposition stage, air temperature, habitat type (open or closed canopy), soil condition (dry or wet soil), and carcass condition (not frozen or previously frozen) on the mean daily carcass temperature. Air temperature (poly.) was additionally included using a second-order orthogonal polynomial. Carcass ID and series ID were included as a nested random effect (variance = 3.337, std. dev. = 1.827). Significant variables are indicated in bold.

	**Estimate**	***SE***	***z*-value**	***p*-value**
**Intercept**	**1.742**	0.542	3.22	**0.001**
Decomposition stage 2 (putrefaction)	0.255	0.230	1.12	0.268
**Decomposition stage 3 (bloated)**	**0.922**	0.372	2.48	**0.013**
**Decomposition stage 4 (post-bloated)**	**1.334**	0.592	2.26	**0.024**
**Decomposition stage 5 (advanced decay)**	**3.063**	0.563	5.44	**<0.001**
Decomposition stage 6 (dry remains)	1.346	1.319	1.02	0.307
**Air temperature**	**0.586**	0.057	10.23	**<0.001**
Air temperature (polynomial)	0.007	0.004	1.78	0.075
**Habitat type: open**	**1.304**	0.484	2.70	**0.007**
Soil condition: wet	-0.271	0.484	-0.56	0.575
Carcass condition: not frozen	0.280	0.591	0.47	0.635
Decomposition stage 2 * Air temperature	0.01	0.072	0.15	0.882
**Decomposition stage 3 * Air temperature**	**0.176**	0.082	2.15	**0.031**
**Decomposition stage 4 * Air temperature**	**0.957**	0.132	7.26	**<0.001**
**Decomposition stage 5 * Air temperature**	**0.636**	0.113	5.61	**<0.001**
Decomposition stage 6 * Air temperature	0.156	0.222	0.70	0.484
**Decomposition stage 2 * Air temp. (poly.)**	**0.013**	0.005	2.59	**0.010**
Decomposition stage 3 * Air temp. (poly.)	0.010	0.005	1.92	0.055
**Decomposition stage 4 * Air temp. (poly.)**	**-0.022**	0.007	-3.18	**0.002**
**Decomposition stage 5 * Air temp. (poly.)**	**-0.012**	0.006	-2.18	**0.029**
Decomposition stage 6 * Air temp. (poly.)	-0.001	0.009	0.10	0.920

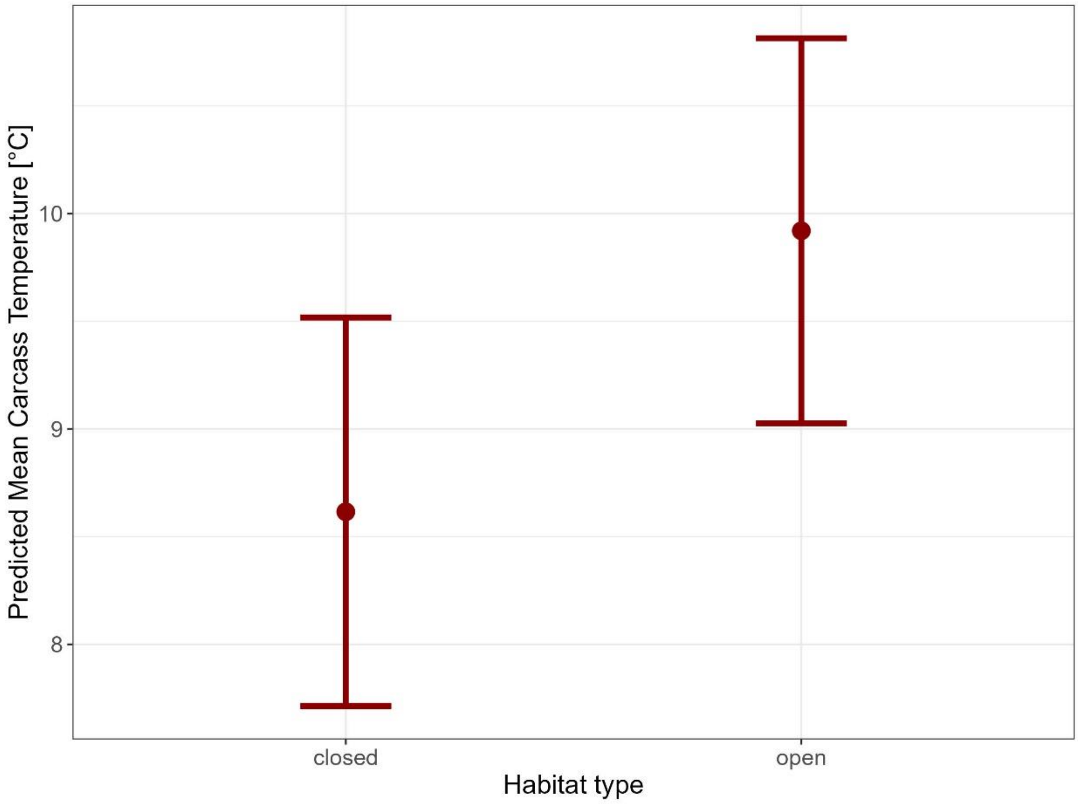

Figure S3: Predicted mean carcass temperatures (°C) for closed and open habitats.

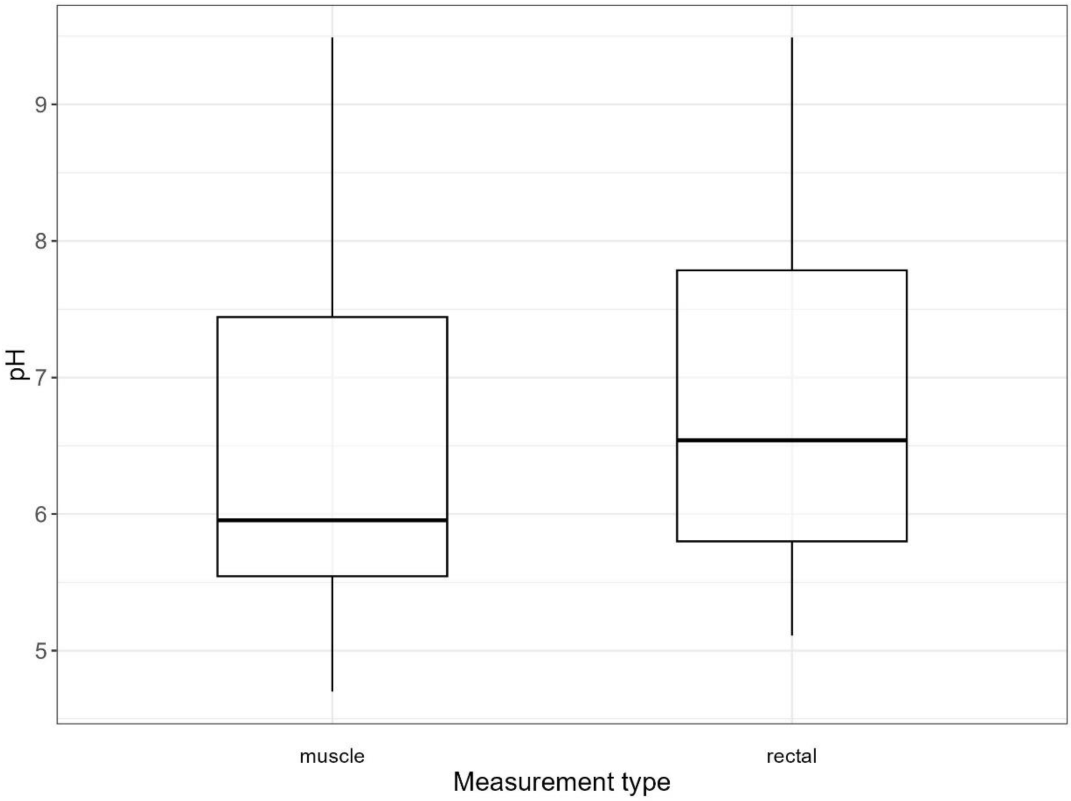

Figure S4: Difference in carcass pH between measurements in the hind thigh muscle and rectum.

Table S4: Effects of decomposition stage and carcass temperature and their interactions on the muscle pH measured in situ in wild boar carcasses. These predictor variables were included as interaction terms. Regression parameters are on the log-scale. Carcass ID was included as a random effect (variance = 0.001, std. dev. = 0.035). Significant variables are indicated in bold.

	Estimate	*SE*	*z*-value	*p*-value
**Intercept**	**1.855**	0.047	37.36	**<0.001**
Decomposition stage 2 (putrefaction)	0.013	0.056	0.24	0.810
**Decomposition stage 3 (bloated)**	**–0.146**	0.073	–2.01	**0.044**
**Decomposition stage 4 (post-bloated)**	**–0.175**	0.078	–2.24	**0.025**
Decomposition stage 5 (advanced decay)	0.109	0.069	1.59	0.112
**Decomposition stage 6 (dry remains)**	**0.433**	0.066	6.58	**<0.001**
Carcass temperature	–0.008	0.004	–1.79	0.073
Decomposition stage 2 * Carcass temperature	–0.000	0.005	–0.09	0.926
**Decomposition stage 3 * Carcass temperature**	**0.012**	0.006	1.98	**0.048**
**Decomposition stage 4 * Carcass temperature**	**0.018**	0.005	3.29	**0.001**
**Decomposition stage 5 * Carcass temperature**	**0.012**	0.005	2.29	**0.022**
Decomposition stage 6 * Carcass temperature	–0.006	0.006	–1.01	0.311

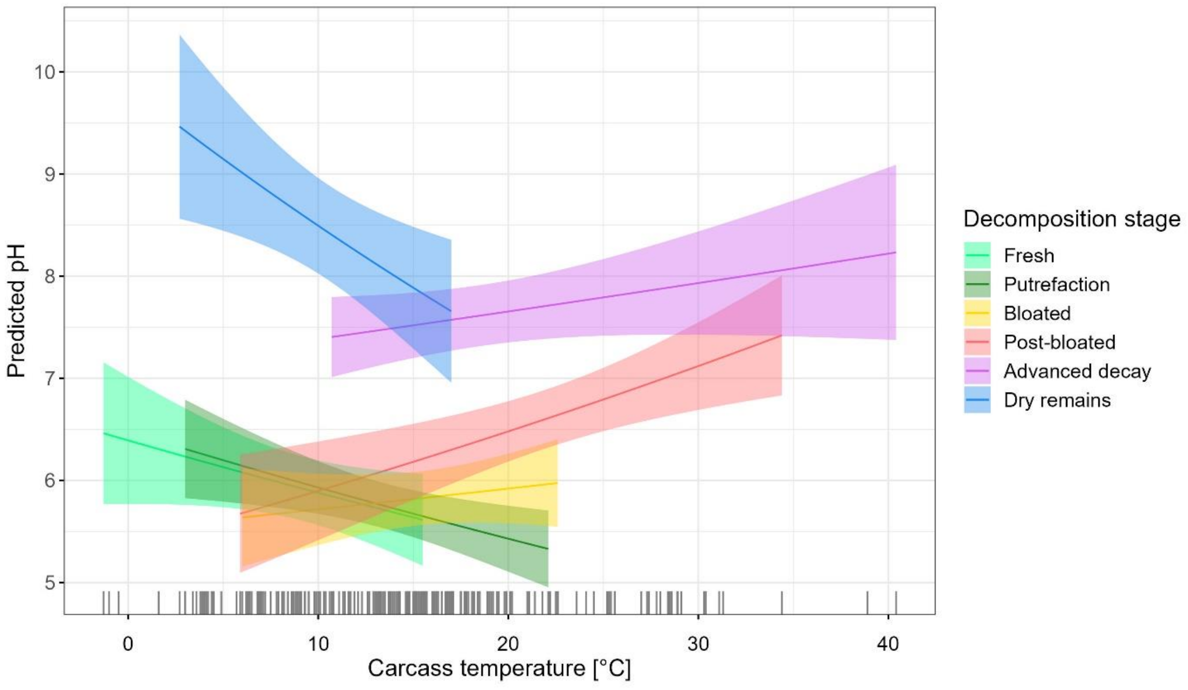

Figure S5: Predicted pH in the hind thigh muscle of decomposing wild boar carcasses for all decomposition stages, considering carcass temperature. Carcass temperatures are highest during the post-bloated and advanced decomposition stages and then decrease with time. The highest pH is reached at the dry remains stage.

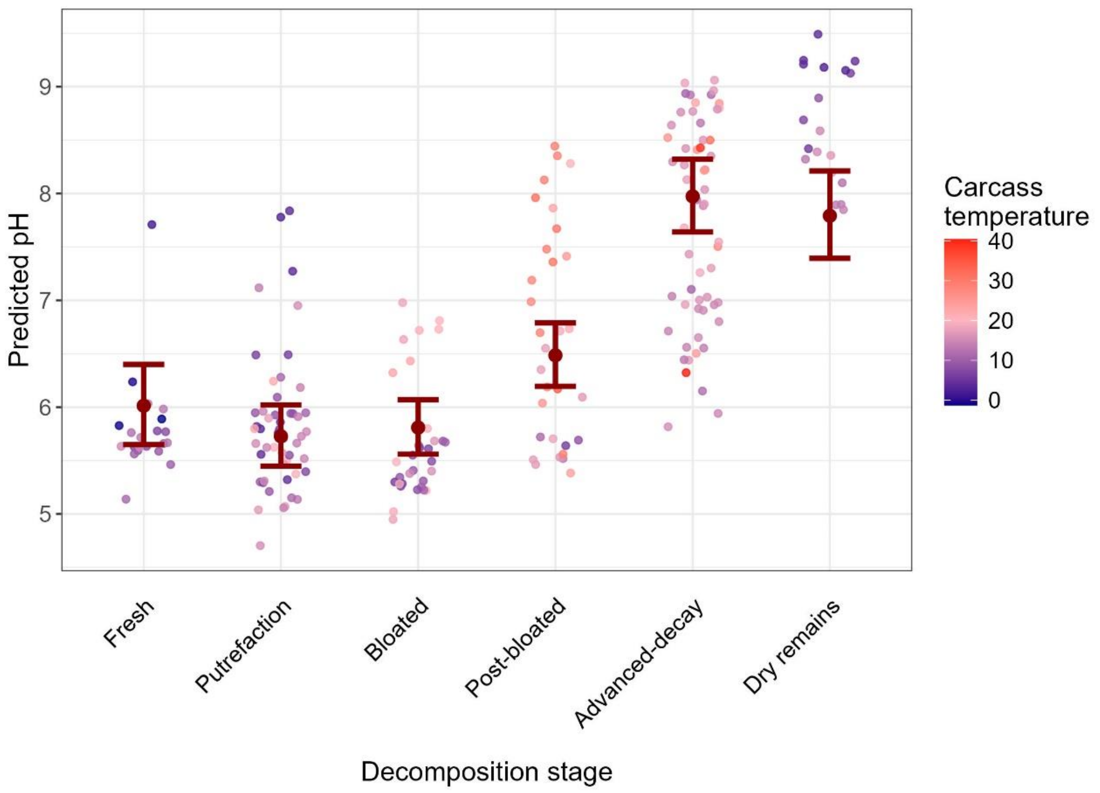

Figure S6: Predicted pH measured rectally in decomposing wild boar carcasses. Points represent the observed values, displayed in the same color as the corresponding carcass temperatures. The highest temperatures were measured during the post-bloated and advanced decay stages.

Table S5: Effect of the decomposition stage and carcass temperature and their interactions on the rectal pH of wild boar carcasses. These predictor variables were included as an interaction term. Regression parameters are on the log-scale. Carcass ID was included as a random effect (variance = 0.003, std. dev. = 0.053). Significant variables are indicated in bold.

	Estimate	*SE*	z-value	*p*-value
**Intercept**	**1.851**	0.037	50.56	**<0.001**
**Decomposition stage 2 (putrefaction)**	**0.146**	0.037	3.92	**<0.001**
**Decomposition stage 3 (bloated)**	**–0.125**	0.049	–2.53	**0.011**
Decomposition stage 4 (post-bloated)	–0.066	0.044	–1.49	0.136
**Decomposition stage 5 (advanced decay)**	**0.177**	0.040	4.45	**<0.001**
Decomposition stage 6 (dry remains)	0.157	0.081	1.94	0.053
Carcass temperature	–0.004	0.003	–1.23	0.220
**Decomposition stage 2 * Carcass temperature**	**–0.012**	0.003	–3.58	**<0.001**
Decomposition stage 3 * Carcass temperature	0.006	0.004	1.47	0.141
**Decomposition stage 4 * Carcass temperature**	**0.009**	0.003	2.77	**0.006**
**Decomposition stage 5 * Carcass temperature**	**0.006**	0.003	2.20	**0.028**
Decomposition stage 6 * Carcass temperature	0.006	0.005	1.29	0.197

Table S6: Parameter estimates of GLMM model 4, evaluating the effects of decomposition stage, soil (wet or dry), and carcass condition (shot and not frozen or previously frozen) on soil pH. Carcass ID and series ID were included as a nested random effect (variance = 0.008, std. dev. = 0.089). Significant variables are indicated in bold.

	Estimate	*SE*	*z*-value	*p*-value
**Intercept**	**1.550**	0.025	62.35	**<0.001**
**Decomposition stage 2 (putrefaction)**	**0.137**	0.020	6.58	**<0.001**
**Decomposition stage 3 (bloated)**	**0.155**	0.027	5.78	**<0.001**
**Decomposition stage 4 (post-bloated)**	**0.273**	0.028	9.85	**<0.001**
**Decomposition stage 5 (advanced decay)**	**0.376**	0.025	14.88	**<0.001**
**Decomposition stage 6 (dry remains)**	**0.447**	0.025	17.74	**<0.001**
Soil condition (wet)	0.040	0.027	1.46	0.145
Carcass condition (not frozen)	–0.010	0.035	–0.29	0.775

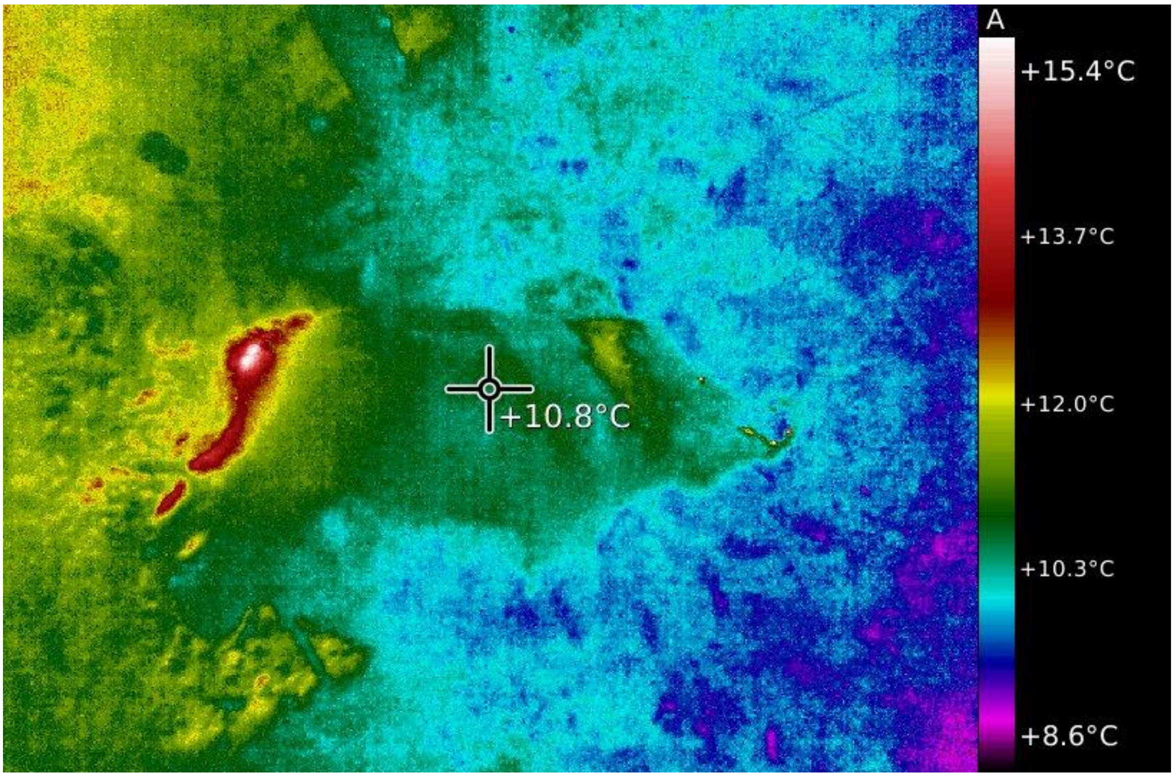

Figure S7: Thermal image of a decomposing wild boar carcass in September, at the onset of decomposition. Insect activity led to the accumulation of maggots at the rectum, visible in bright red and white.
