# Supplementary material for: Temperature and pH dynamics during carcass decomposition and implications for disease management
Source: Sci Rep. 2025 Jul 16;15:25795. doi: 10.1038/s41598-025-07716-w (PMC12267392; doi:10.1038/s41598-025-07716-w)
Supplement: Supplementary file 2 — Supplementary Material 2 [file 41598_2025_7716_MOESM2_ESM.docx]

Supplementary material

Temperature and pH dynamics during carcass decomposition and implications for disease management

Janine Rietz, Burkhard Beudert, Nicolas Ferry, Lukas Böcker, Franz J. Conraths, Carolina Probst, Andreas Zedrosser, Helmut Küchenhoff, Martin Hais, Jens Schlüter, Tomas Lackner, Christian von Hoermann, Jörg Müller, Marco Heurich


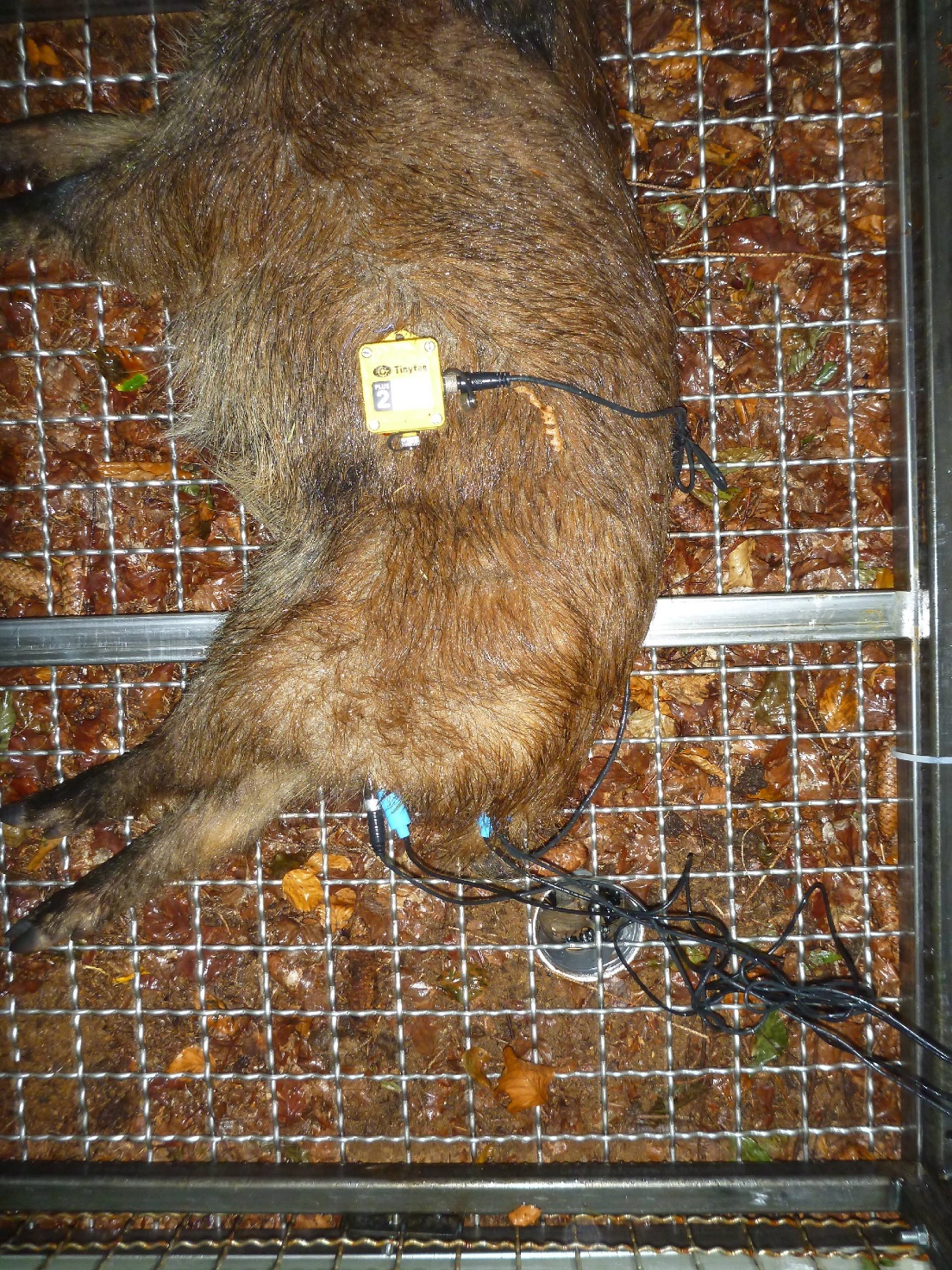


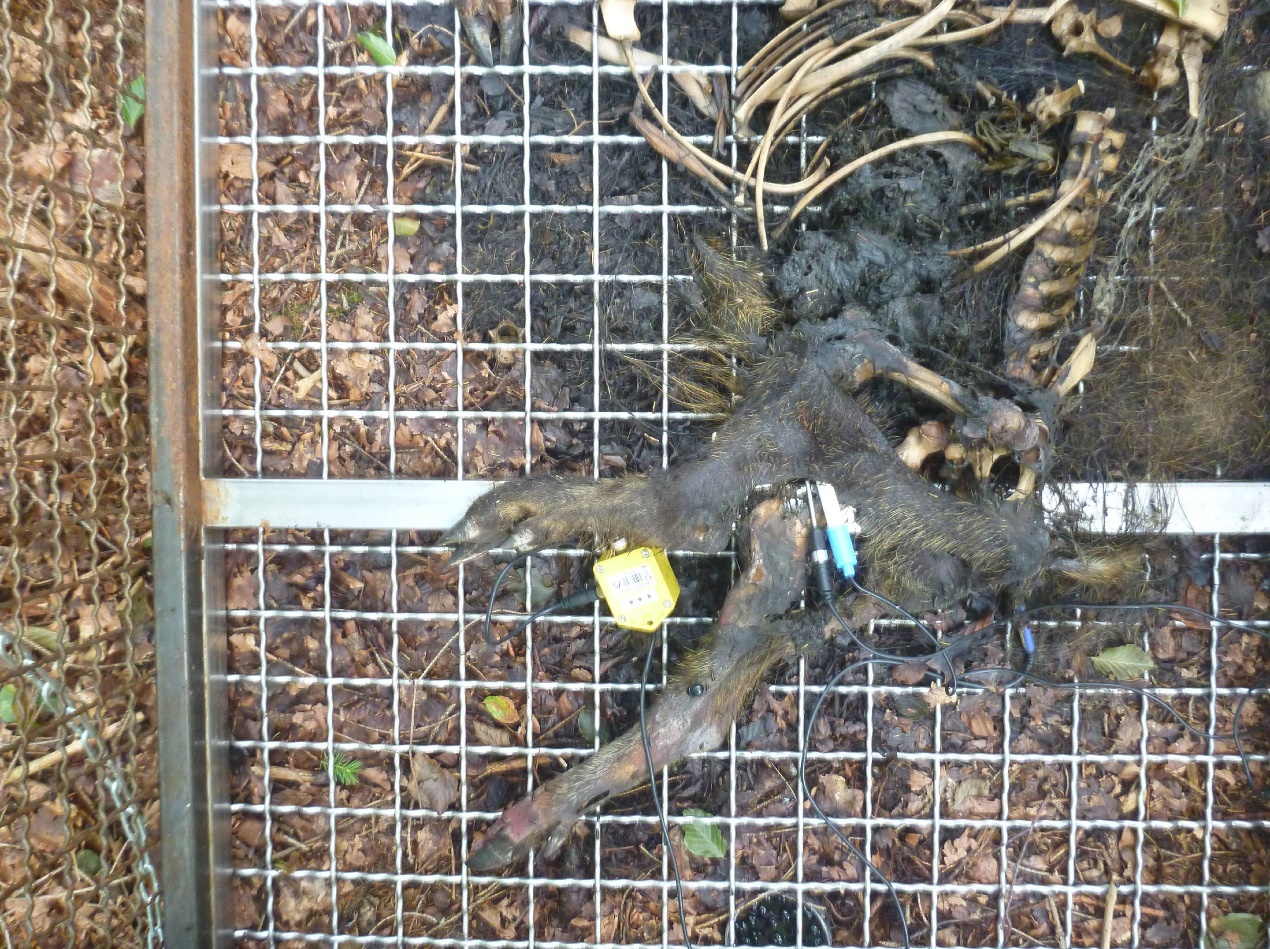


Figure S1: pH and temperature probes inserted rectally and into the hind thigh muscle of two wild boar carcasses placed in cages. The upper photo shows a carcass shortly after placement, with initial pH and temperature measurements. The lower photo shows a carcass at the onset of skeletonization (beginning of the dry remains stage), marking the end of the field experiment. The pH and temperature probes, previously positioned between the skin and bones, were removed shortly thereafter.


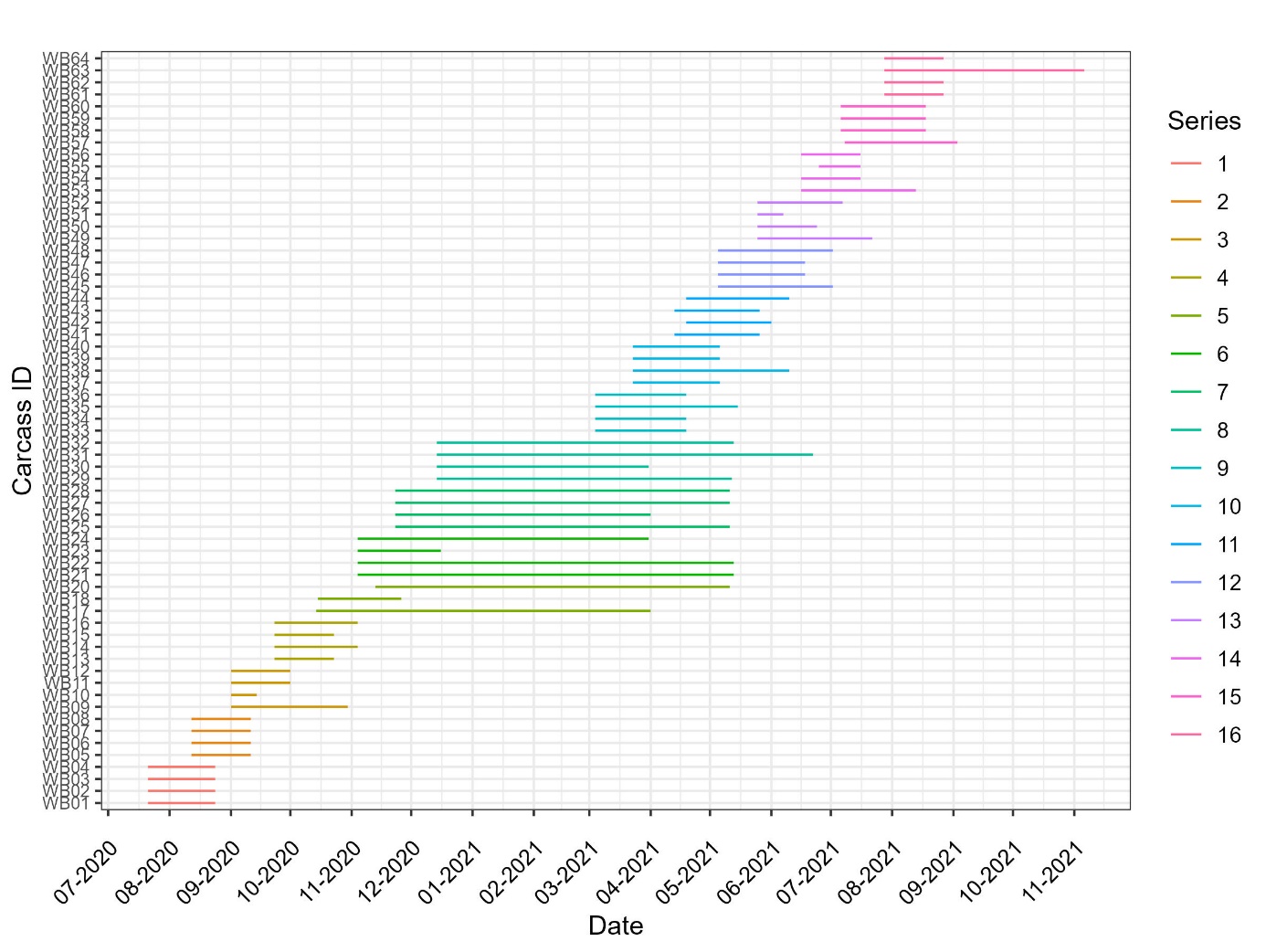


Figure S2: Time span of carcass monitoring and measurements for each carcass, grouped by placement series.

Table S1: Overview of the environmental and carcass-related variables as well as the number of sampling days for each carcass/plot ID. Plot ID labels: WB = wild boar, CC = carcass in cage. Fresh refers to freshly shot and not frozen (yes) or frozen (no) carcasses. Sampling days indicate the number of soil samples taken.

| **Series ID** | **Date** | **Plot ID** | **Fresh** | **Sex** | **Weight (kg)** | **Habitat** | **Soil** | **Sampling days** |
| --- | --- | --- | --- | --- | --- | --- | --- | --- |
| 1 | 21.07.2020 | WB01 | yes | male | 28.8 | open | wet | 10 |
| 1 | 21.07.2020 | WB02 | no | female | 41.0 | open | dry | 10 |
| 1 | 21.07.2020 | WB03 | no | female | 28.0 | closed | wet | 10 |
| 1 | 21.07.2020 | WB04 | no | female | 29.5 | closed | dry | 10 |
| 1 | 21.07.2020 | CC01 | no | male | 33.5 | closed | dry | 9 |
| 2 | 12.08.2020 | WB05 | no | male | 36.0 | open | dry | 9 |
| 2 | 12.08.2020 | WB06 | yes | female | 13.6 | closed | dry | 3 |
| 2 | 12.08.2020 | WB07 | no | female | 45.5 | open | wet | 3 |
| 2 | 12.08.2020 | WB08 | no | female | 9.4 | closed | wet | 3 |
| 2 | 12.08.2020 | CC02 | no | male | 30.0 | closed | dry | 8 |
| 3 | 01.09.2020 | WB09 | no | female | 74.0 | closed | dry | 4 |
| 3 | 01.09.2020 | WB10 | no | male | 10.0 | open | wet | 9 |
| 3 | 01.09.2020 | WB11 | yes | female | 9.0 | closed | wet | 4 |
| 3 | 01.09.2020 | WB12 | no | male | 49.5 | open | dry | 4 |
| 3 | 01.09.2020 | CC03 | no | male | 21.5 | closed | dry | 9 |
| 4 | 23.09.2020 | WB13 | no | male | 16.0 | open | wet | 4 |
| 4 | 23.09.2020 | WB14 | no | female | 50.0 | closed | wet | 13 |
| 4 | 23.09.2020 | WB15 | yes | male | 19.3 | closed | dry | 4 |
| 4 | 23.09.2020 | WB16 | no | female | 38.0 | open | dry | 4 |
| 4 | 23.09.2020 | CC04 | no | male | 24.5 | closed | dry | 9 |
| 5 | 13.10.2020 | WB17 | no | female | 48.0 | closed | dry | 17 |
| 5 | 13.10.2020 | WB18 | yes | male | 25.5 | open | wet | 5 |
| 5 | 13.10.2020 | WB19 | no | male | 20.0 | open | dry | 5 |
| 5 | 13.10.2020 | WB20 | no | female | 22.0 | closed | wet | 8 |
| 5 | 13.10.2020 | CC05 | no | male | 49.0 | closed | dry | 14 |
| 6 | 04.11.2020 | WB21 | no | female | 21.6 | open | wet | 8 |
| 6 | 04.11.2020 | WB22 | yes | female | 29.7 | closed | dry | 8 |
| 6 | 04.11.2020 | WB23 | no | female | 76.0 | closed | wet | 7 |
| 6 | 04.11.2020 | WB24 | no | male | 11.5 | open | dry | 6 |
| 6 | 04.11.2020 | CC06 | no | male | 43.0 | closed | dry | 9 |
| 7 | 23.11.2020 | WB25 | no | female | 37.0 | open | wet | 10 |
| 7 | 23.11.2020 | WB26 | no | male | 59.0 | closed | dry | 6 |
| 7 | 23.11.2020 | WB27 | no | female | 73.0 | open | dry | 9 |
| 7 | 23.11.2020 | WB28 | no | female | 25.5 | closed | wet | 10 |
| 8 | 14.12.2020 | WB29 | yes | male | 62.0 | closed | wet | 4 |
| 8 | 14.12.2020 | WB30 | no | female | 38.5 | open | dry | 8 |
| 8 | 14.12.2020 | WB31 | no | male | 75.0 | closed | dry | 7 |
| 8 | 14.12.2020 | WB32 | no | male | 58.0 | open | wet | 4 |
| 9 | 03.03.2021 | WB33 | no | male | 37.0 | open | dry | 15 |
| 9 | 03.03.2021 | WB34 | yes | male | 43.0 | closed | dry | 15 |
| 9 | 03.03.2021 | WB35 | no | male | 58.3 | closed | wet | 15 |
| 9 | 03.03.2021 | WB36 | no | female | 32.5 | open | wet | 7 |
| **Series ID** | **Date** | **Plot ID** | **Fresh** | **Sex** | **Weight (kg)** | **Habitat** | **Soil** | **Sampling days** |
| 10 | 23.03.2021 | WB37 | no | female | 50.0 | closed | wet | 13 |
| 10 | 23.03.2021 | WB38 | no | female | 68.0 | closed | dry | 14 |
| 10 | 23.03.2021 | WB39 | no | female | 41.8 | open | wet | 5 |
| 10 | 23.03.2021 | WB40 | yes | male | 33.8 | open | dry | 6 |
| 11 | 13.04.2021 | WB41 | yes | male | 58.5 | open | wet | 5 |
| 11 | 13.04.2021 | WB42 | no | female | 74.0 | closed | dry | 5 |
| 11 | 13.04.2021 | WB43 | no | male | 54.0 | open | dry | 5 |
| 11 | 13.04.2021 | WB44 | no | male | 60.0 | closed | wet | 11 |
| 11 | 13.04.2021 | CC07 | no | female | 60.0 | closed | dry | 11 |
| 12 | 05.05.2021 | WB45 | yes | male | 57.5 | open | dry | 11 |
| 12 | 05.05.2021 | WB46 | no | female | 54.0 | closed | wet | 8 |
| 12 | 05.05.2021 | WB47 | no | female | 45.0 | open | wet | 5 |
| 12 | 05.05.2021 | WB48 | no | male | 44.5 | closed | dry | 8 |
| 13 | 25.05.2021 | WB49 | no | male | 62.0 | open | dry | 4 |
| 13 | 25.05.2021 | WB50 | no | female | 58.0 | closed | dry | 4 |
| 13 | 25.05.2021 | WB51 | no | male | 45.7 | closed | wet | 2 |
| 13 | 25.05.2021 | WB52 | yes | male | 56.0 | open | wet | 10 |
| 14 | 16.06.2021 | WB53 | no | female | 44.0 | open | wet | 4 |
| 14 | 16.06.2021 | WB54 | no | male | 55.5 | open | dry | 5 |
| 14 | 16.06.2021 | WB55 | yes | male | 93.0 | closed | wet | 4 |
| 14 | 16.06.2021 | WB56 | no | male | 50.0 | closed | dry | 9 |
| 14 | 16.06.2021 | CC08 | no | male | 48.0 | closed | dry | 5 |
| 15 | 06.07.2021 | WB57 | yes | male | 57.0 | closed | wet | 10 |
| 15 | 06.07.2021 | WB58 | no | male | 40.2 | closed | dry | 4 |
| 15 | 06.07.2021 | WB59 | no | female | 35.0 | open | dry | 4 |
| 15 | 06.07.2021 | WB60 | no | male | 56.0 | open | wet | 4 |
| 15 | 06.07.2021 | CC09 | no | male | 29.6 | closed | dry | 8 |
| 16 | 27.07.2021 | WB61 | no | male | 45.0 | closed | wet | 4 |
| 16 | 27.07.2021 | WB62 | no | female | 54.0 | open | dry | 10 |
| 16 | 27.07.2021 | WB63 | yes | male | 95.0 | closed | dry | 4 |
| 16 | 27.07.2021 | WB64 | no | male | 53.0 | open | wet | 4 |
| 16 | 27.07.2021 | CC10 | no | male | 50.0 | closed | dry | 9 |

Table S2: Number of carcasses and number of events (nEvents) above specific carcass temperature thresholds (30–55°C) lasting 30, 60, 90 min, and > 90 min. The median, mean, and maximum durations (in minutes) of these events are also reported. Due to sampling frequency, the minimum duration was consistently 30 min. Temperatures > 45°C were reached only during aerobic decomposition (stages 4 - post-bloated, 5 - advanced decay, and 6 - dry remains), while temperatures between 35 and 45°C were also reached during stage 2 -putrefaction and stage 3 - bloated.

| **Carcass temperature (°C)** | **Number of carcasses** | **nEvents 30 min** | **nEvents 60 min** | **nEvents 90 min** | **nEvents > 90 min** | **Median (min)** | **Mean**  **(min)** | **Maximum**  **(min)** |
| --- | --- | --- | --- | --- | --- | --- | --- | --- |
| **55** | 2 | 2 | 0 | 1 | 1 | 60 | 68 | 120 |
| **50** | 7 | 2 | 4 | 1 | 6 | 90 | 129 | 330 |
| **45** | 12 | 10 | 8 | 7 | 18 | 90 | 112 | 300 |
| **40** | 23 | 40 | 18 | 10 | 62 | 90 | 176 | 2700 |
| **35** | 31 | 57 | 34 | 24 | 141 | 150 | 312 | 4530 |
| **30** | 41 | 72 | 50 | 23 | 236 | 210 | 451 | 7020 |

Table S3: Parameter estimates of generalized linear Model 1, evaluating the effect of decomposition stage, air temperature, habitat type (open or closed canopy), soil condition (dry or wet soil), and carcass condition (not frozen or previously frozen) on the mean daily carcass temperature. Air temperature (poly.) was additionally included using a second-order orthogonal polynomial. Carcass ID and series ID were included as a nested random effect (variance = 3.337, std. dev. = 1.827). Significant variables are indicated in bold.

|  | Estimate | *SE* | *z*-value | *p*-value |
| --- | --- | --- | --- | --- |
| Intercept | **1.742** | 0.542 | 3.22 | **0.001** |
| Decomposition stage 2 (putrefaction) | 0.255 | 0.230 | 1.12 | 0.268 |
| Decomposition stage 3 (bloated) | **0.922** | 0.372 | 2.48 | **0.013** |
| Decomposition stage 4 (post-bloated) | **1.334** | 0.592 | 2.26 | **0.024** |
| Decomposition stage 5 (advanced decay) | **3.063** | 0.563 | 5.44 | **<0.001** |
| Decomposition stage 6 (dry remains) | 1.346 | 1.319 | 1.02 | 0.307 |
| Air temperature | **0.586** | 0.057 | 10.23 | **<0.001** |
| Air temperature (polynomial) | 0.007 | 0.004 | 1.78 | 0.075 |
| Habitat type: open | **1.304** | 0.484 | 2.70 | **0.007** |
| Soil condition: wet | -0.271 | 0.484 | -0.56 | 0.575 |
| Carcass condition: not frozen | 0.280 | 0.591 | 0.47 | 0.635 |
| Decomposition stage 2 * Air temperature | 0.01 | 0.072 | 0.15 | 0.882 |
| Decomposition stage 3 * Air temperature | **0.176** | 0.082 | 2.15 | **0.031** |
| Decomposition stage 4 * Air temperature | **0.957** | 0.132 | 7.26 | **<0.001** |
| Decomposition stage 5 * Air temperature | **0.636** | 0.113 | 5.61 | **<0.001** |
| Decomposition stage 6 * Air temperature | 0.156 | 0.222 | 0.70 | 0.484 |
| Decomposition stage 2 * Air temp. (poly.) | **0.013** | 0.005 | 2.59 | **0.010** |
| Decomposition stage 3 * Air temp. (poly.) | 0.010 | 0.005 | 1.92 | 0.055 |
| Decomposition stage 4 * Air temp. (poly.) | **-0.022** | 0.007 | -3.18 | **0.002** |
| Decomposition stage 5 * Air temp. (poly.) | **-0.012** | 0.006 | -2.18 | **0.029** |
| Decomposition stage 6 * Air temp. (poly.) | -0.001 | 0.009 | 0.10 | 0.920 |


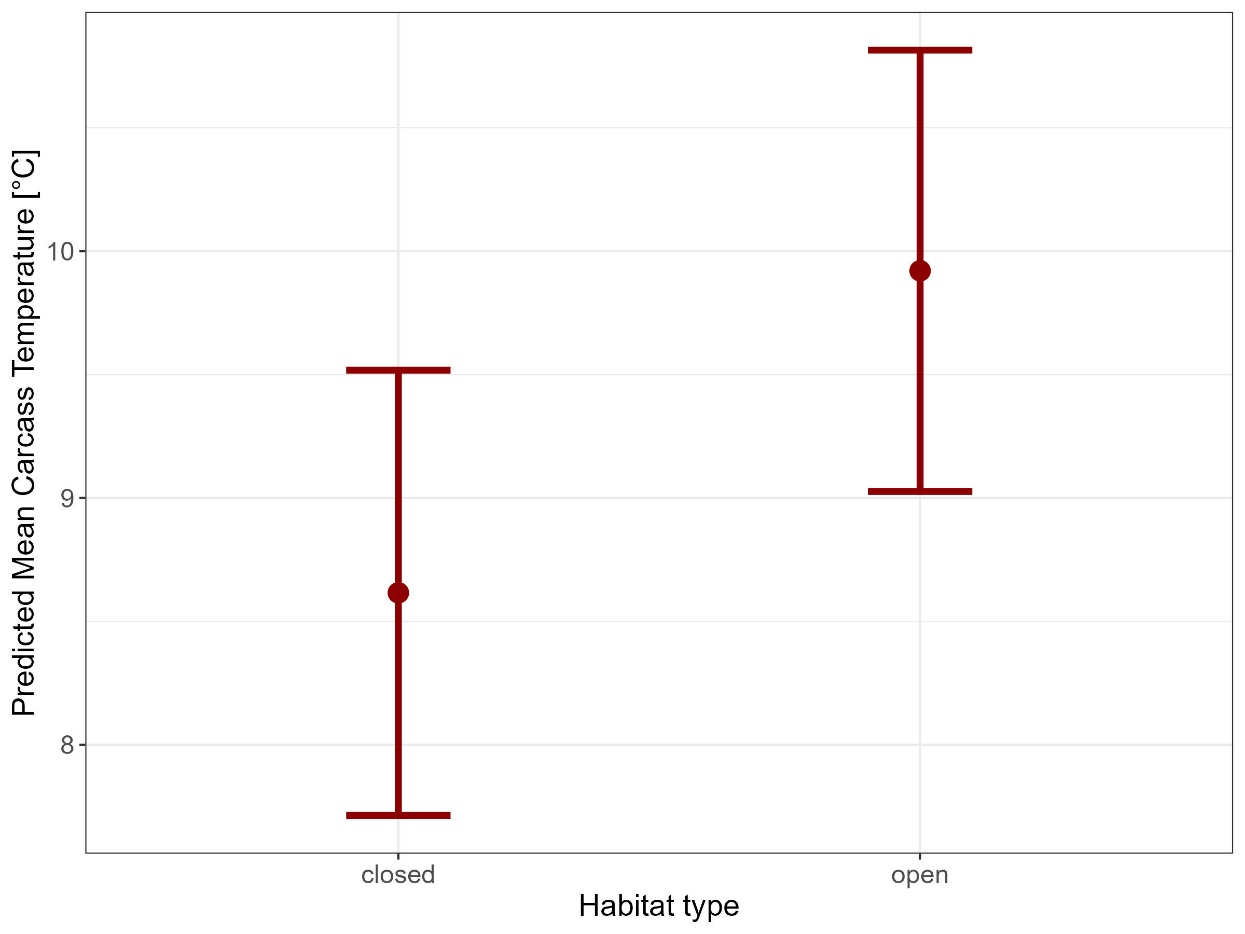


Figure S3: Predicted mean carcass temperatures (°C) for closed and open habitats.


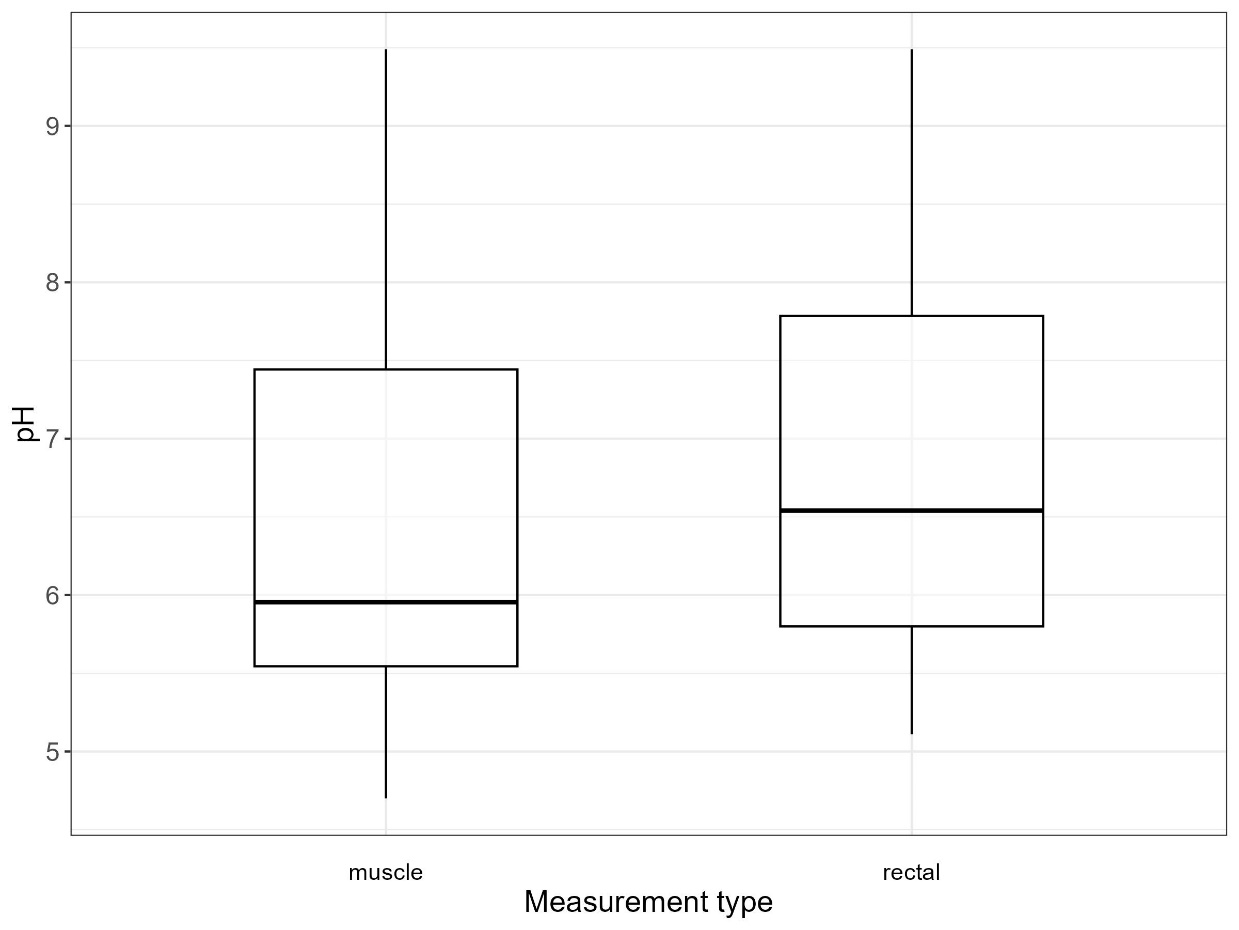


Figure S4: Difference in carcass pH between measurements in the hind thigh muscle and rectum.

Table S4: Effects of decomposition stage and carcass temperature and their interactions on the muscle pH measured in situ in wild boar carcasses. These predictor variables were included as interaction terms. Regression parameters are on the log-scale. Carcass ID was included as a random effect (variance = 0.001, std. dev. = 0.035). Significant variables are indicated in bold.

|  | Estimate | *SE* | *z*-value | *p*-value |
| --- | --- | --- | --- | --- |
| Intercept | **1.855** | 0.047 | 37.36 | **<0.001** |
| Decomposition stage 2 (putrefaction) | 0.013 | 0.056 | 0.24 | 0.810 |
| Decomposition stage 3 (bloated) | **–0.146** | 0.073 | –2.01 | **0.044** |
| Decomposition stage 4 (post-bloated) | **–0.175** | 0.078 | –2.24 | **0.025** |
| Decomposition stage 5 (advanced decay) | 0.109 | 0.069 | 1.59 | 0.112 |
| Decomposition stage 6 (dry remains) | **0.433** | 0.066 | 6.58 | **<0.001** |
| Carcass temperature | –0.008 | 0.004 | –1.79 | 0.073 |
| Decomposition stage 2 * Carcass temperature | –0.000 | 0.005 | –0.09 | 0.926 |
| Decomposition stage 3 * Carcass temperature | **0.012** | 0.006 | 1.98 | **0.048** |
| Decomposition stage 4 * Carcass temperature | **0.018** | 0.005 | 3.29 | **0.001** |
| Decomposition stage 5 * Carcass temperature | **0.012** | 0.005 | 2.29 | **0.022** |
| Decomposition stage 6 * Carcass temperature | –0.006 | 0.006 | –1.01 | 0.311 |


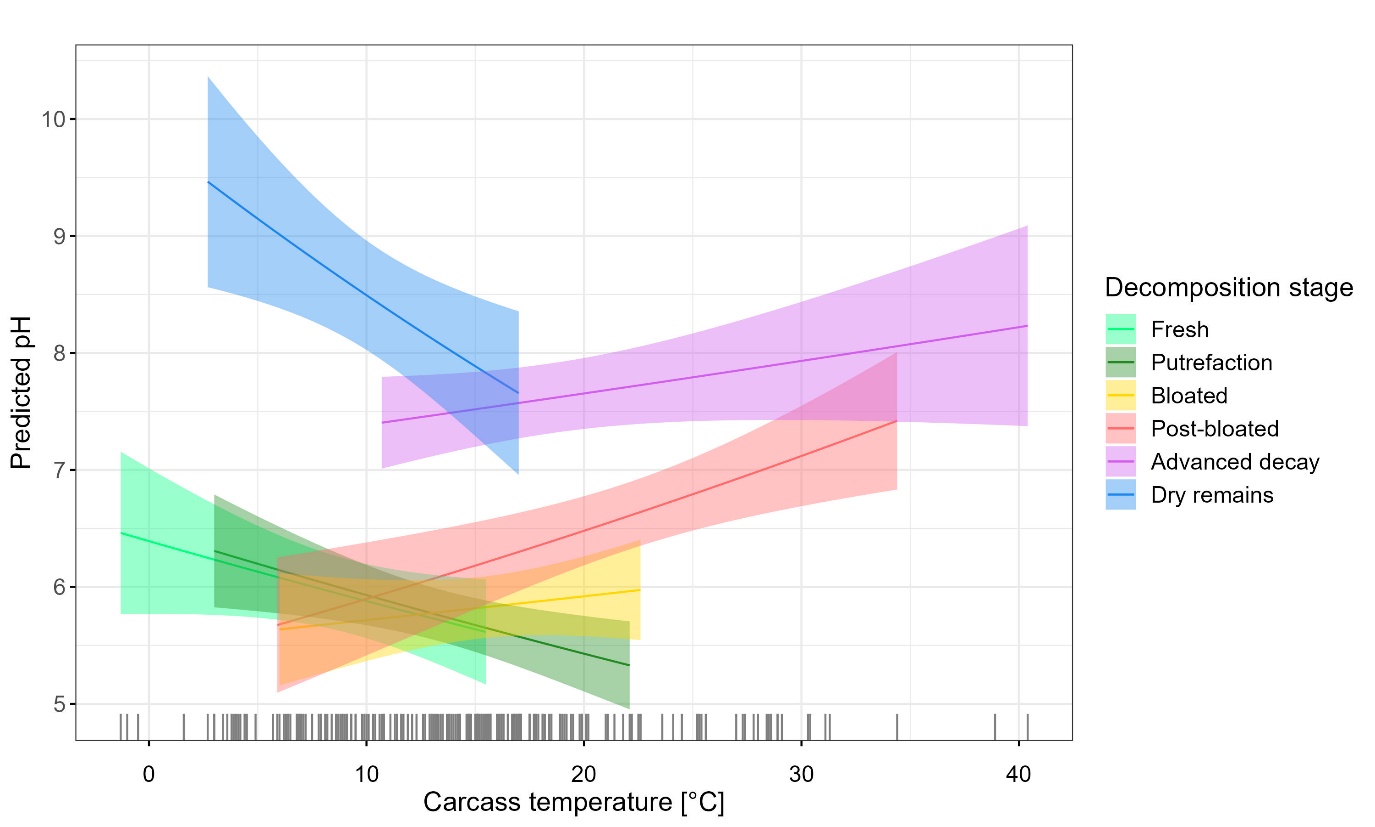


Figure S5: Predicted pH in the hind thigh muscle of decomposing wild boar carcasses for all decomposition stages, considering carcass temperature. Carcass temperatures are highest during the post-bloated and advanced decomposition stages and then decrease with time. The highest pH is reached at the dry remains stage.


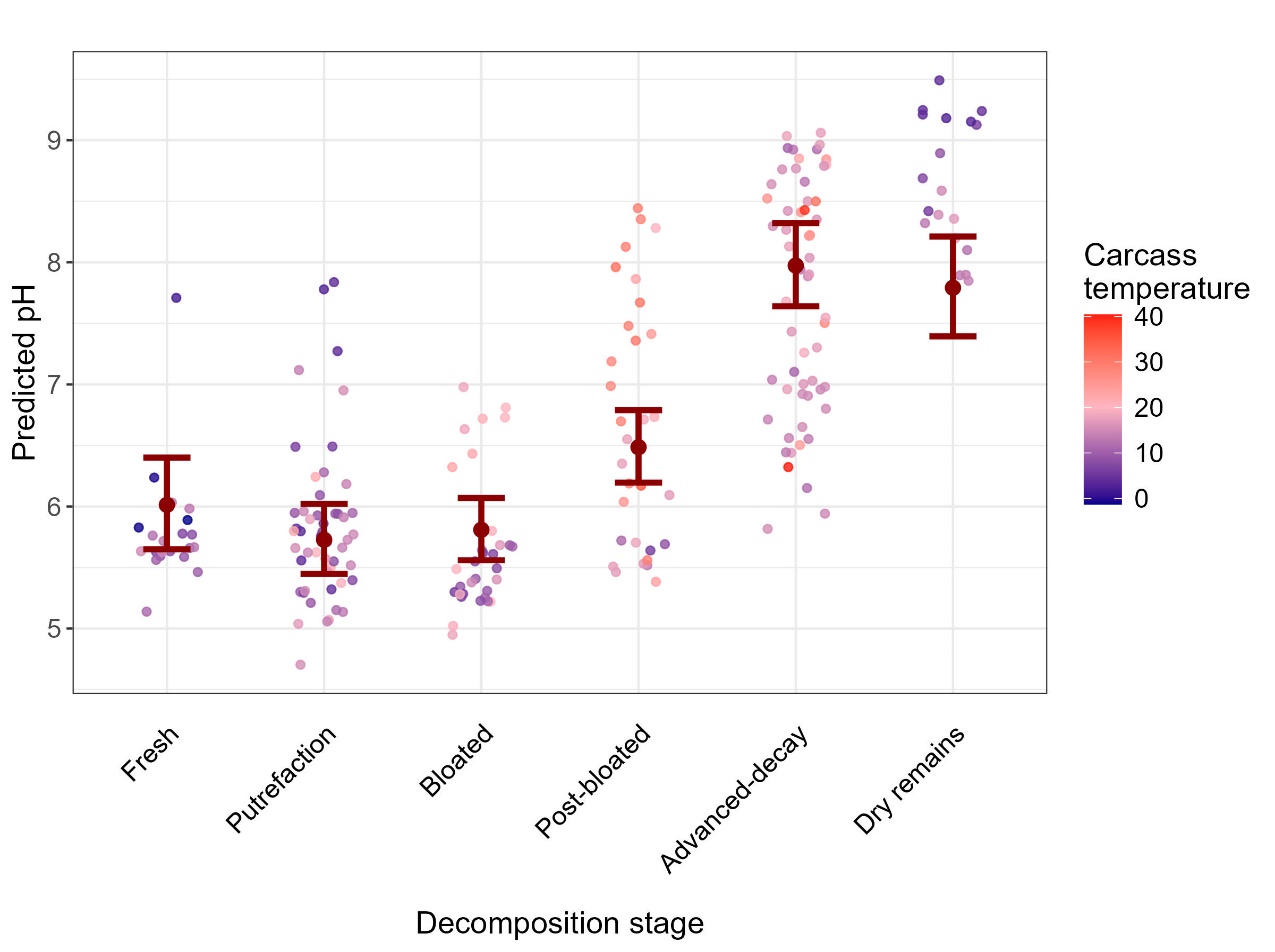


Figure S6: Predicted pH measured rectally in decomposing wild boar carcasses. Points represent the observed values, displayed in the same color as the corresponding carcass temperatures. The highest temperatures were measured during the post-bloated and advanced decay stages.

Table S5: Effect of the decomposition stage and carcass temperature and their interactions on the rectal pH of wild boar carcasses. These predictor variables were included as an interaction term. Regression parameters are on the log-scale. Carcass ID was included as a random effect (variance = 0.003, std. dev. = 0.053). Significant variables are indicated in bold.

|  | Estimate | *SE* | z-value | *p*-value |
| --- | --- | --- | --- | --- |
| Intercept | **1.851** | 0.037 | 50.56 | **<0.001** |
| Decomposition stage 2 (putrefaction) | **0.146** | 0.037 | 3.92 | **<0.001** |
| Decomposition stage 3 (bloated) | **–0.125** | 0.049 | –2.53 | **0.011** |
| Decomposition stage 4 (post-bloated) | –0.066 | 0.044 | –1.49 | 0.136 |
| Decomposition stage 5 (advanced decay) | **0.177** | 0.040 | 4.45 | **<0.001** |
| Decomposition stage 6 (dry remains) | 0.157 | 0.081 | 1.94 | 0.053 |
| Carcass temperature | –0.004 | 0.003 | –1.23 | 0.220 |
| Decomposition stage 2 * Carcass temperature | **–0.012** | 0.003 | –3.58 | **<0.001** |
| Decomposition stage 3 * Carcass temperature | 0.006 | 0.004 | 1.47 | 0.141 |
| Decomposition stage 4 * Carcass temperature | **0.009** | 0.003 | 2.77 | **0.006** |
| Decomposition stage 5 * Carcass temperature | **0.006** | 0.003 | 2.20 | **0.028** |
| Decomposition stage 6 * Carcass temperature | 0.006 | 0.005 | 1.29 | 0.197 |

Table S6: Parameter estimates of GLMM model 4, evaluating the effects of decomposition stage, soil (wet or dry), and carcass condition (shot and not frozen or previously frozen) on soil pH. Carcass ID and series ID were included as a nested random effect (variance = 0.008, std. dev. = 0.089). Significant variables are indicated in bold.

|  | Estimate | *SE* | *z*-value | *p*-value |
| --- | --- | --- | --- | --- |
| Intercept | **1.550** | 0.025 | 62.35 | **<0.001** |
| Decomposition stage 2 (putrefaction) | **0.137** | 0.020 | 6.58 | **<0.001** |
| Decomposition stage 3 (bloated) | **0.155** | 0.027 | 5.78 | **<0.001** |
| Decomposition stage 4 (post-bloated) | **0.273** | 0.028 | 9.85 | **<0.001** |
| Decomposition stage 5 (advanced decay) | **0.376** | 0.025 | 14.88 | **<0.001** |
| Decomposition stage 6 (dry remains) | **0.447** | 0.025 | 17.74 | **<0.001** |
| Soil condition (wet) | 0.040 | 0.027 | 1.46 | 0.145 |
| Carcass condition (not frozen) | –0.010 | 0.035 | –0.29 | 0.775 |


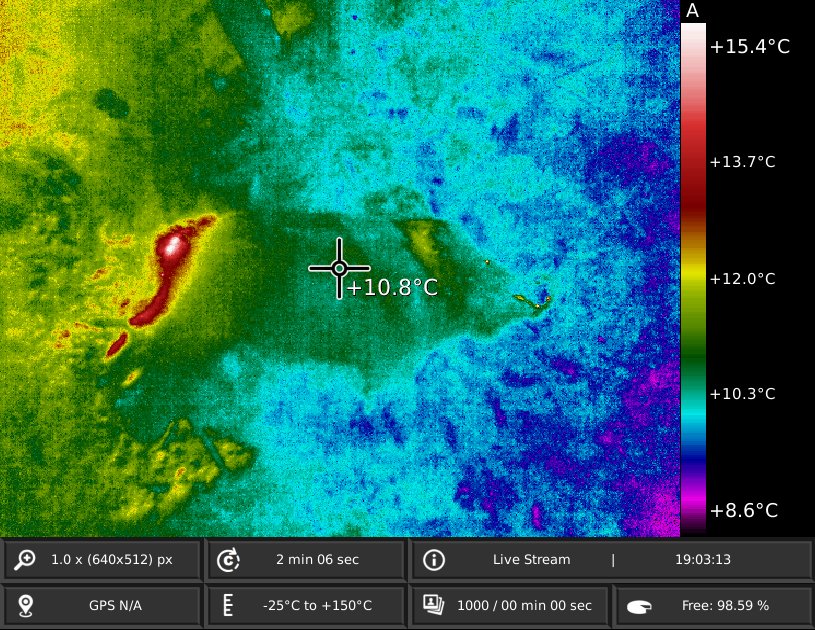


Figure S7: Thermal image of a decomposing wild boar carcass in September, at the onset of decomposition. Insect activity led to the accumulation of maggots at the rectum, visible in bright red and white.
